# A causal association between obesity and constipation: a two-sample bidirectional Mendelian randomization study and meta-analysis

**DOI:** 10.3389/fnut.2024.1430280

**Published:** 2024-11-11

**Authors:** Xian Sun, Shuoqiu Zhang, Xi Zhou

**Affiliations:** ^1^School of Integrative Medicine, Nanjing University of Chinese Medicine, Nanjing, China; ^2^Jiangsu Research Center for Chinese Medicine Development, Nanjing, China

**Keywords:** obesity, constipation, Mendelian randomization, meta-analysis, causal association

## Abstract

**Objective:**

Observational studies suggest a potential link between obesity and constipation, but existing results are conflicting. Therefore, we conducted a Mendelian randomization (MR) study and meta-analysis to assess the causal relationship between obesity and the risk of constipation.

**Methods:**

In this study, independent genetic variants closely related to constipation were acquired from a genome-wide association study (GWAS) to analyze the relationship between genetically predisposed obesity and the risk of constipation. Waist circumference (WC), hip circumference (HC), waist-to-hip ratio (WHR), and body mass index (BMI) were collected from the GWAS. Then, the causal relationship between constipation and obesity was explored using a two-sample MR study in both directions. The robustness of the results was evaluated using sensitivity analysis. Furthermore, a systemic review and meta-analysis were performed to calculate relative risks (RRs) with corresponding 95% confidence intervals (95% CIs). Subgroup analyses stratified by age and obesity degree were completed. To evaluate whether the current studies were affected by unmeasured confounders, *E*-values of each study were determined.

**Results:**

In MR analysis, the incidence of constipation increased with the increase in BMI [inverse variance-weighted (IVW) odds ratio (OR) = 1.138 (1.029, 1.260), *p* = 0.012]. In addition, constipation was impacted by WC [IVW OR = 1.220 (1.061, 1.402), *p* = 0.005]. However, there was no evidence that WHR [IVW OR = 1.833 (0.826, 4.065), *p* = 0.136] or HC [IVW OR = 0.949, (0.836, 1.077), *p* = 0.415] has a causal effect on constipation. In reverse MR analysis, there was no evidence supporting the causality between constipation and obesity [BMI IVW OR = 1.010 (0.998, 1.022), *p* = 0.089; WHR IVW OR = 1.000 (0.946, 1.057), *p* = 0.994; WC IVW OR = 1.008 (0.995, 1.022), *p* = 0.217; HC IVW OR = 0.996 (0.982, 1.011), *p* = 0.626]. In the meta-analysis, 14 eligible articles were included, involving 43,488 subjects. According to the results of the meta-analysis, the risk of obesity and overweight significantly increased the risk of constipation [RR = 1.145 (0.952, 1.376)]. This was consistent with the MR analysis results. Moreover, overweight and obesity were significantly related to a higher constipation risk among children [overweight RR = 1.112 (0.943, 1.312); obesity RR = 1.407 (1.282, 1.544)]. Additionally, overweight in adults could decrease the risk of constipation [RR = 0.940 (0.827, 1.068)]. Nevertheless, no significant association was observed between obesity in adults and the risk of constipation [RR = 1.000 (0.768, 1.303)]. Sensitivity analysis revealed the robustness of our findings.

**Conclusion:**

In this combined MR study and meta-analysis, obesity is associated with an increased risk of constipation. The MR analysis demonstrates the causal relationship between genetically predisposed obesity and the risk of constipation. More research is required to investigate the potential correlation between obesity and the risk of constipation and associated mechanisms.

## 1 Introduction

Constipation, a common gastrointestinal disorder globally, is reported to influence 4 and 10.2% of men and women, respectively (1). This effect includes physical and social functioning and mental health of patients (2, 3). The cost of medication for constipation patients is very expensive. In the United States, the annual medical expenses of constipation treatment reach US$230 million (4). Constipation is associated with many factors. As reported in one cross-sectional study in Turkey, physical inactivity, decreased water/fiber consumption, aging, female sex, and obesity are related to the higher constipation risk (5).

Obesity is a health problem worldwide. Globally, the incidence of obesity and its associated disorders exhibits an increasing trend, and there are currently over 2 billion overweight people (6). In addition, its morbidity and mortality are widely investigated in different organ systems, such as its gastrointestinal presentations on the gallbladder, liver, and upper gastrointestinal motility (7, 8).

The association between obesity and constipation has attracted significant attention, with increasing evidence indicating a complex interaction between the two. Constipation and obesity have not been associated statistically significantly in children (9), while some studies suggest that obesity might contribute to adult constipation (10). However, several critical gaps and unresolved issues need to be further investigated. Current research predominantly concentrates on a unidirectional approach, assessing how obesity increases the risk of constipation (11). There has been limited exploration of how constipation may influence obesity in return and potentially exacerbate it. This unidirectional design may overlook potential bidirectional causal relationships, causing an incomplete understanding of the interactive mechanisms between the two conditions. For example, a study performed by Costa et al. (12) indicated a significant association between obesity and constipation through data analysis but could not determine whether this relationship was causal or driven by other factors. Obesity and constipation are influenced by many factors, including age, sex, and genetic background, which vary across different populations. Sample heterogeneity in various studies has contributed to inconsistent findings.

Bidirectional Mendelian randomization uses genetic variants as instrumental variables, leveraging the random allocation of genetic variants to mimic randomized controlled trials. This approach effectively controls confounders and helps uncover the causal relationship between obesity and constipation, clarifying whether obesity directly leads to constipation or whether constipation influences obesity in return. The application of bidirectional MR has greatly benefited from advances in large-scale genome-wide association studies, providing high-quality genetic instruments for MR analyses. Using the latest statistical methods, the precision and reliability of the results can be improved, enhancing the clinical translation potential of this approach. Different from traditional unidirectional MR, bidirectional MR not only assesses the causal effect of obesity on constipation but also explores the reverse effect of constipation on obesity. Moreover, this innovative bidirectional analysis offers a comprehensive view of disease interaction pathways, providing a more complete scientific basis for developing clinical treatment and prevention strategies. Moreover, the combination of MR analysis and meta-analysis can significantly enhance the persuasive of results. Therefore, the present MR study and meta-analysis were performed to investigate the causal association between obesity and the risk of constipation.

## 2 Materials and methods

### 2.1 Mendelian randomization analysis

#### 2.1.1 Study design

**Figure 1:**
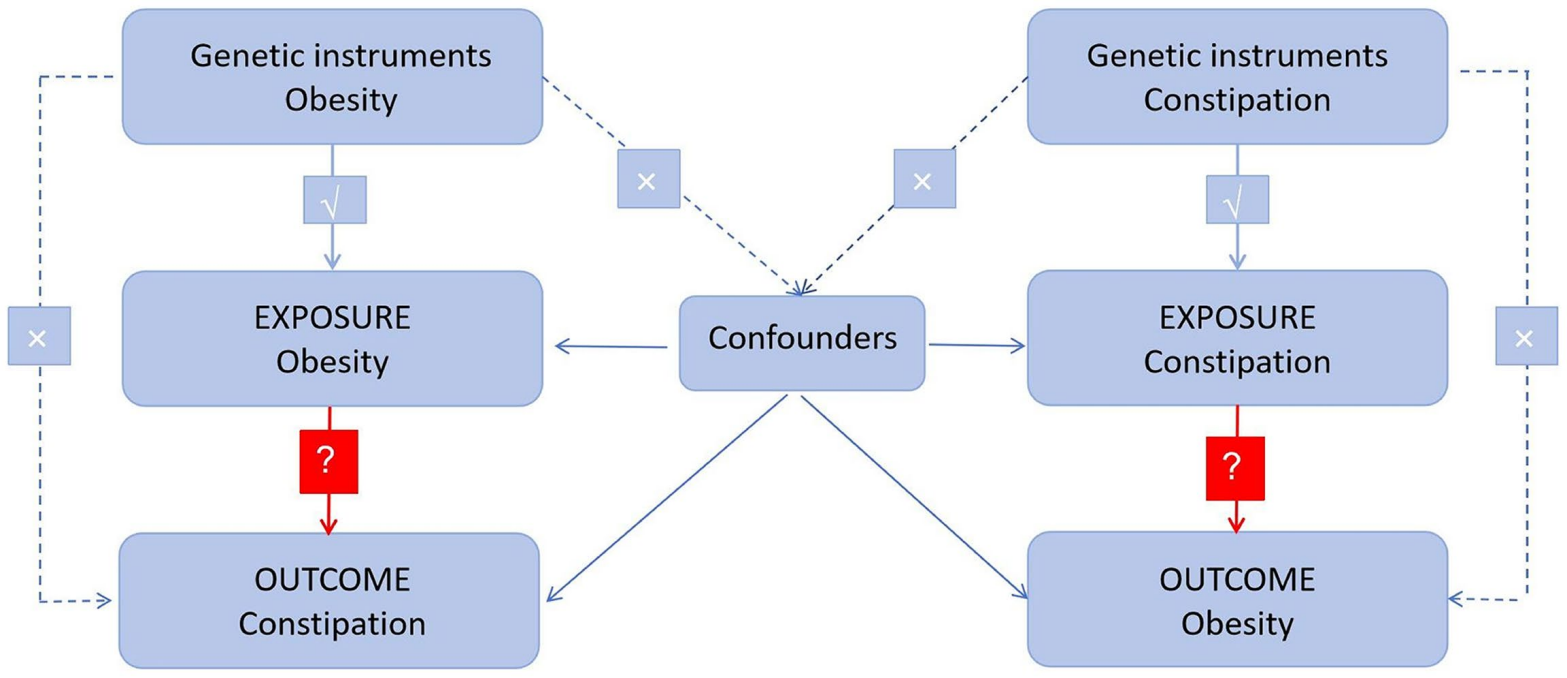
Diagram of critical assumptions for MR analysis. The solid line indicates that genetic instruments (SNPs) are related to exposure and can only affect the results through exposure. The dashed line indicates genetic instruments (SNPs) independent of any confounding variables between the results.

This two-sample MR analysis used several single nucleotide polymorphisms (SNPs) representing genetic variation. There are three assumptions to be satisfied (Figure 1): (1) instrumental variables (IVs) are associated with exposure; (2) IVs are independent of other confounders; and (3) genetic variation influences outcomes solely through exposure (13). In addition, a bidirectional causal association between obesity and constipation was evaluated using MR analysis.

#### 2.1.2 Data sources

In adults, overweight was deemed as body mass index (BMI) ≥25 kg/m^2^, while obesity was deemed as BMI ≥ 30 kg/m^2^ based on the criteria of the World Health Organization (WHO). In adolescents, as defined by the growth charts of the Centers for Disease Control and Prevention, overweight and obesity were classified as the 85th–95th and ≥ 95th percentiles in age- and sex-specific BMI distributions, respectively (14). BMI has been the common method used globally for measuring body fat and thinness degree. The general obesity index is calculated according to the BMI [total mass (kg)/height square (m^2^)]. WHR [WC (cm)/HC (cm)] and IBM-adjusted WHR are considered the central obesity indices. We searched BMI (*n* = 532,396), WHR (*n* = 142,762), WC (*n* = 462,166), and HC (*n* = 336,601) as exposure factors in European ancestry, which were derived from GWASs, such as the IEU open GWAS, and the GWAS Catalog, and obtained qualified datasets. Additionally, BMI (*n* = 158,284) and HC (*n* = 92,615) GWAS summary data from East Asian ancestry could be acquired using the same method.

Single nucleotide polymorphisms that were obtained from the GWAS dataset show significance at the whole genome level (*p* < 5 × 10^−8^) and were selected for this study. Ethical approval was waived due to the use of data from public databases.

In total, 451,567 subjects of European ancestry were enrolled in our constipation analysis, including 4,781 participants with constipation and 44,6,786 healthy controls. A total of 176,629 subjects of East Asian ancestry were included, consisting of 397 participants with constipation and 176,232 healthy controls. Supplementary Table S1 presents the exposure and outcome features. There existed no overlapping population in GWASs between outcomes and exposures.

#### 2.1.3 SNP screening

Single nucleotide polymorphisms are frequently seen as genetic variants in human beings, which can serve as IVs to replace traits in MR analysis (15, 16). Appropriate SNPs were selected from the exposure-related GWASs based on the following criteria: (1) a genome-wide significance *p*-value of <5 × 10^−8^; if sufficient SNPs were not available, a relaxed threshold of 5 × 10^−6^ was used. (2) *F*-statistics >10. (3) SNPs in linkage disequilibrium (LD) were eliminated using a clumping window of 10 MB and *r*^2^ value <0.01. (4) PhenoScanner V2 was applied to eliminate SNPs associated with outcomes and potential confounders.

#### 2.1.4 Two-sample Mendelian randomization

This MR study operated in two opposite directions, with one predicting the causal impact of obesity on constipation, whereas the other predicted the effect of constipation on obesity.

In this study, the inverse variance weighted (IVW) method was used as the main MR method as it is the earliest and the most commonly used method in MR analysis (17). Meanwhile, four other MR methods, namely, weighted median, weighted mode, simple mode, and MR-Egger were applied. Among them, the IVW method conducts MR on the impact of SNP exposure on the outcome, with adjustment for heterogeneity. By contrast, the weighted median determines the median causal estimate, the weighted mode determines the mode, and the simple mode predicts causal association with no weights, while the MR-Egger addresses the pleiotropy problem. The integration of these approaches can strengthen the robustness of the results and provide various insights. In IVW analysis, valid IVs are assumed, and deviations influence the precision. These methods contribute to comprehensively viewing the causality, considering different assumptions and biases. In this study, the MR study was carried out using the TwoSampleMR package (version: 0.5.8) and R Software (version: 4.3.2).

#### 2.1.5 Heterogeneity and sensitivity analyses

A fixed-effect IVW approach was used for primary analysis to analyze the overall effects without heterogeneity (18). Nevertheless, a random-effects model IVW approach was used when heterogeneity was determined between causal estimates of genetic variances. Cochran’s *Q* statistic was calculated in the MR study to predict the presence/absence of heterogeneity, with a *p* value <0.05 indicating the existence of heterogeneity in the MR analysis. The fixed-effects IVW method was used when the *p-*value was >0.05 in Cochran’s *Q* statistic, while the random-effects IVW approach was utilized when the *p* value was <0.05 (19). *I*^2^statistic was determined to explore the heterogeneity level (20).

Sensitivity analysis was carried out to examine whether the MR results were robust against possible deviation from three major assumptions. A *p* value of MR-Egger regression’s intercept was used to assess horizontal pleiotropy. If pleiotropy exists (*p* < 0.05), outliers of SNPs were removed. A leave-one-out test was used to estimate the potential pleiotropy effect of single SNPs.

### 2.2 Meta-analysis

#### 2.2.1 Study retrieval

**Figure 2:**
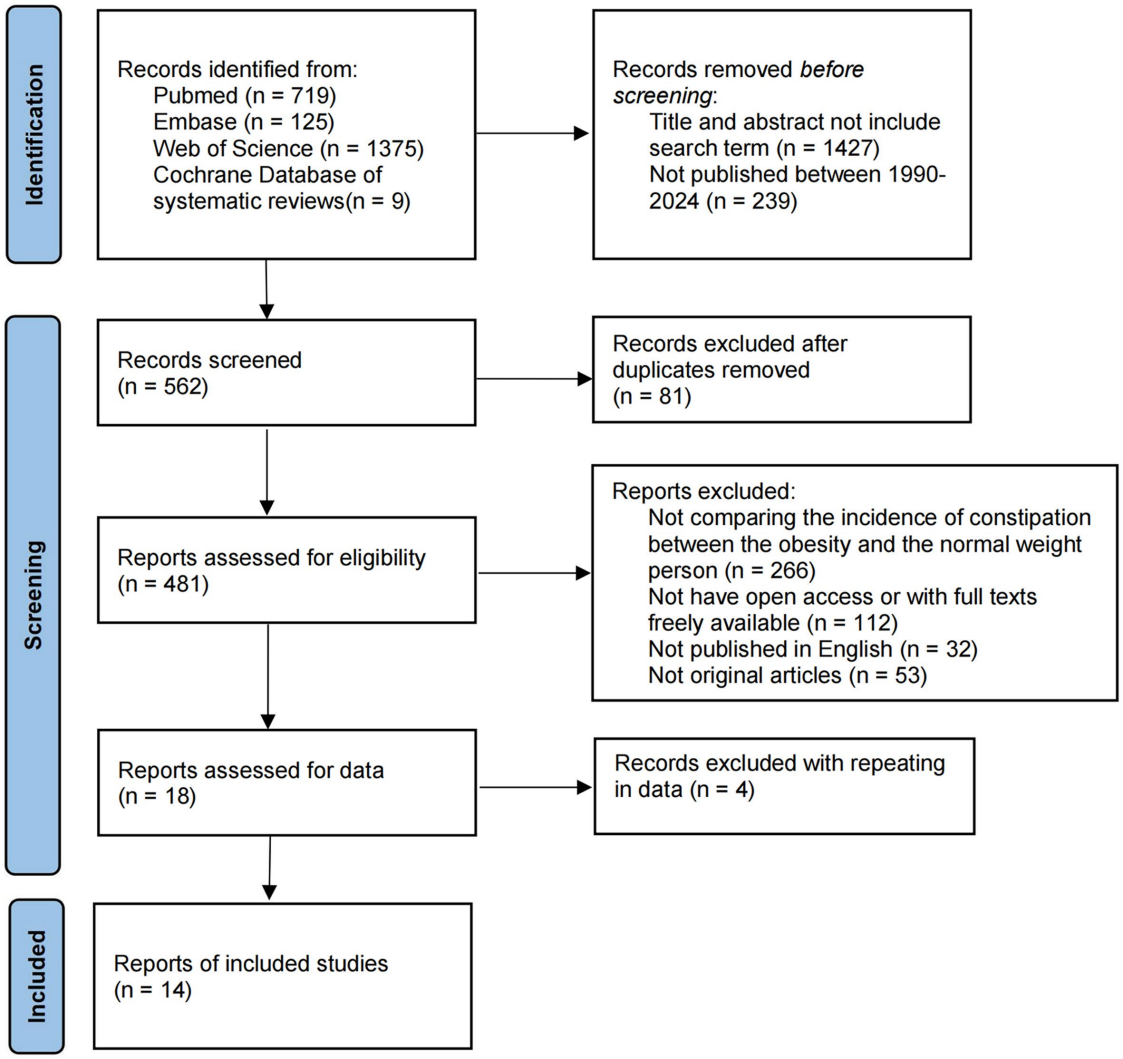
Flow diagram detailing the search strategy and identification of studies used in meta-analysis.

The Cochrane Library, Web of Science, PubMed, and Embase databases were searched using the string “((overweight) OR obesity) AND constipation” from inception to 1 February 2024 to identify relevant studies. Supplementary Table S5 displays search strings used for identifying studies from the PubMed, Cochrane Library, Embase, and Web of Science databases. Figure 2 presents the study retrieval process. In addition, this study also manually searched references in eligible articles to prevent the omission of qualified articles. We would contact with original authors to obtain further information.

Publications concerning constipation risk in obese patients satisfying the criteria mentioned below were enrolled: (1) cohort or case–control or cross-sectional studies; (2) articles assessing the association between obesity and constipation risk; and (3) articles with available or calculable relative risk (RR) or odds ratio (OR) and 95% CIs.

The following publications were eliminated: (1) case reports; (2) articles from referral centers; (3) non-English publications, duplicates, or conference abstracts with no follow-up publication; and (4) studies with unavailable data to calculate effect size for this meta-analysis.

#### 2.2.2 Data extraction and quality assessment

Data were extracted and validated by two authors (Shuoqiu Zhang and Xian Sun), with disputes resolved through discussions or the opinion of a third author (Xi Zhou). The data extracted from each included publication consisted of the last name of first author, publication year, study region/country, participant number (cases and controls/non-cases/cohort size), study quality, and study design. For at-risk cases and non-at-risk cases/participants, their data were adjusted for different exposure categories and covariates prior to later analysis, if necessary.

#### 2.2.3 Bias risk evaluation

Bias risk was assessed by two authors (Shuoqiu Zhang, and Xian Sun) independently according to the description in the Cochrane Handbook, and any dispute between them was settled through discussion. Randomization generation, allocation concealment, blinding, patient proportion completing follow-up, intention-to-treat analysis, and selective outcome reporting were recorded.

#### 2.2.4 Quality assessment

This study employed the Newcastle–Ottawa Quality Assessment Scale to assess cohort study and case–control study quality. Any dispute was settled through discussion. The overall scores of 0.0–6.0 and 6.5–9.0 indicated low- and high-quality studies, respectively (21). Cross-sectional studies were evaluated using the 11-item checklist of the Agency for Healthcare Research and Quality (AHRQ) (22), with 0–3, 4–7, and 8–11 points suggesting low, moderate, and high-quality studies, respectively (23). Study quality was evaluated independently by two researchers (Shuoqiu Zhang and Xian Sun).

#### 2.2.5 Statistical analysis

The random-effects model was used in data pooling (24), aiming to provide the conserved estimate of the impact of overweight or obesity, and inter-study heterogeneity was allowed. This study used RRs as the risk estimate. Owing to the low absolute constipation risk in human beings, we deemed ORs as RRs (25, 26). Both Cochran’s Q test and *I*^2^-statistic were used for assessing the possible heterogeneity of our enrolled articles (20, 27). *I*^2^ > 50% and *p* < 0.05 represented heterogeneity in results (28), and thus, a random-effects model should be used; or else, a fixed-effects model would be used. In sensitivity analysis, one study was eliminated each time to evaluate its impact on the pooled risk estimates. Subgroup analyses stratified by child and adult populations were performed. Publication bias was assessed by Egger (29) linear regression. *p* < 0.05 stood for statistical significance. R Software (Version: 4.3.2) and meta package (Version: 6.5-0) were used in statistical analysis.

#### 2.2.6 Ethical statement

This meta-analysis was carried out on the basis of prior publications, without any original data. Therefore, neither patient consent nor ethical approval was needed.

## 3 Results

### 3.1 Mendelian randomization analysis

#### 3.1.1 Causal effects of obesity on constipation

The TwoSampleMR package in R was used for the MR analysis, with constipation as the outcome variable and BMI, WHR, WC, and HC as the exposure variables. The overall forest diagram of the analysis results was presented as follows, focusing on the IVW algorithm. We removed confounding factors rs12765337 and rs6702519, which were associated with lifestyles, including drinking, smoking, and stress, as they might simultaneously influence the occurrence of obesity and constipation.

**Figure 3:**
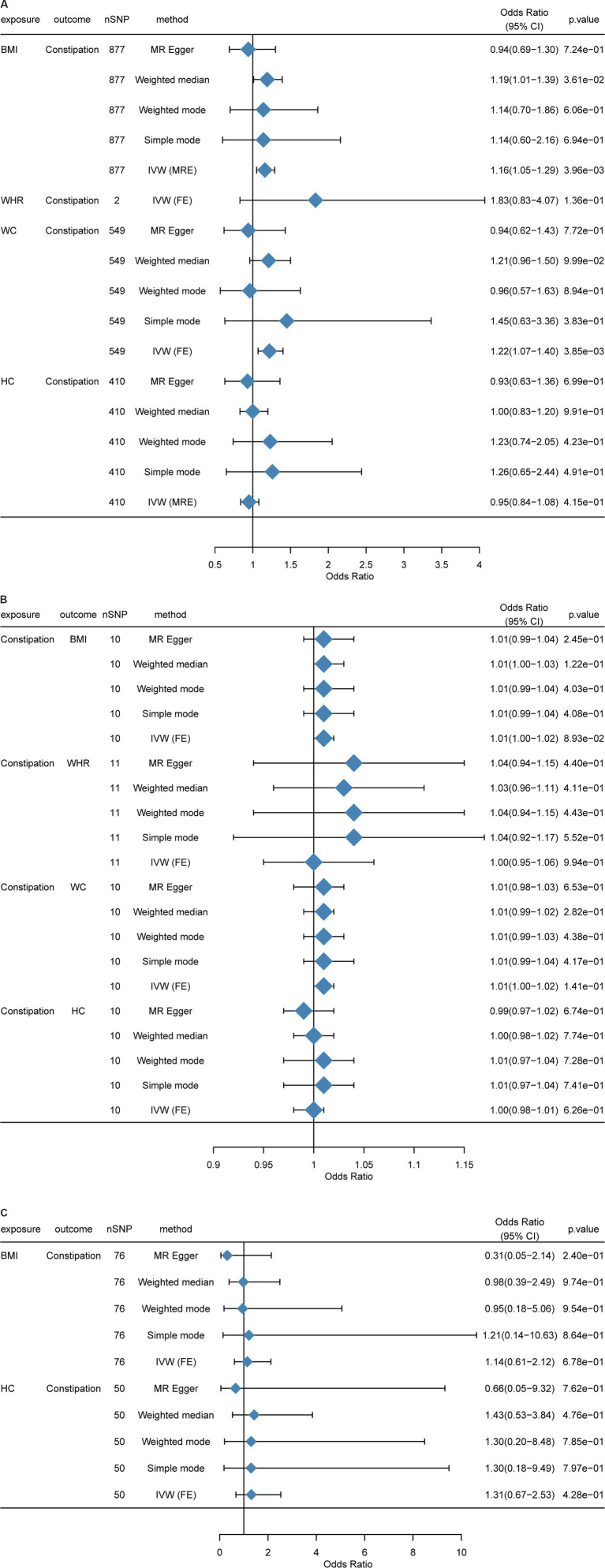
Mendelian randomization between BMI, HC, WC, WHR, and constipation. **(A)** Causal effects of BMI, HC, WC, and WHR on constipation in a European population. **(B)** Causal effects of constipation on BMI, HC, WC, and WHR in a European population. **(C)** Causal effects of BMI, HC, WC, and WHR on constipation in an East Asian population. MR-Egger, weighted median, inverse variance weighted, simple mode, and weighted mode estimates of Mendelian randomization (MR) are summarized. CI, Confidence interval; nSNP, Number of single nucleotide polymorphism; OR, Odds ratio; BMI, Body mass index; HC, Hip circumference; WC, Waist circumference; WHR, Waist-to-hip ratio.

At first, we performed an MR analysis on the European population. As WHR had too few SNPs in common with constipation after *p* value filtering (only two), only the IVW model was used, and there were five models for other indicators. Clearly, BMI and WC were positively related to constipation risk, and the result was significant (*p* < 0.05). Although HC was negatively related to constipation risk, WHR was positively associated with constipation risk, while the results were not significant (*p* > 0.05) (Figure 3A).

Impact of BMI on constipation: In total, 898 independent genome-wide significant SNPs were obtained from GWAS. The SNPs used for the MR study were strong IVs, with each of them having an *F*-statistic >10. In the *F*-statistic, the precision and magnitude of the effect of SNP on BMI were considered. F-statistics of the enrolled individuals were 27-1396. In IVW analysis, BMI was causally related to constipation risk [OR = 1.138 (1.029, 1.260); *p* = 0.012] (Supplementary Table S2; Supplementary Figures S1A–C).

Impact of WHR on constipation: The GWAS obtained two independent genome-wide significant SNPs. The SNPs used for the MR study were “strong” IVs, and all of them had *F*-statistics >10. In the *F*-statistic, the precision and magnitude of the effect of SNP on WHR were considered. The IVW analysis did not detect the causal association (Supplementary Table S2; Supplementary Figures S4A–C). However, limited by SNP number, causal effects between WHR and constipation need more experiments.

Impact of WC on constipation: We obtained 570 independent SNPs with genome-wide significance from GWAS. The SNPs used for the MR study were “strong” IVs, and all of them had F-statistics >10. In the F-statistic, the precision and magnitude of the effect of SNP on WC were considered. The IVW method identified a potential causal association of WC with constipation risk [OR = 1.220 (1.061, 1.402); *p* = 0.005] (Supplementary Table S2; Supplementary Figures S3A–C).

Impact of HC on constipation: We acquired 416 independent SNPs with genome-wide significance from GWAS. The SNPs used for the MR study were “strong” IVs, and all of them had *F*-statistics >10. In the *F*-statistic, the magnitude of the effect of SNP on HC and precision was considered. Moreover, no causal association was identified by IVW analysis (Supplementary Table S2; Supplementary Figures S2A–C).

Because the above analyses were all conducted in the European population, we sincerely aim to replicate this analysis in other populations. However, it is regrettable that we just found East Asian GWAS data for MR analysis. When compared with the European population, the result showed significant differences that BMI [OR = 1.1408 (0.613, 2.123); *p* = 0.677] and HC [OR = 1.306 (0.675, 2.526); *p* = 0.428] were not associated with constipation (Figure 3C). Another result in the Asian population can be found in Supplementary Table S4 and Supplementary Figures S9A–C, S10A–C.

#### 3.1.2 Causal effects of constipation on obesity

The same as the causal effects of obesity and constipation, in this sector, we also used R language to perform MR analysis, with BMI, WHR, WC, and HC as the outcome variables and constipation as the exposure variable. Due to the small amount of data, the threshold of the *p* value was set to 5 × 10^−6^ (30). It can be found that all the results of reverse MR were not significant (*p* > 0.05); that is, constipation did not cause significant changes in obesity-related indicators (Figure 3B).

##### 3.1.2.1 Impact of constipation on BMI

There were 10 independent SNPs with genome-wide significance obtained from GWAS. The SNPs utilized for the MR study were “strong” IVs whose *F*-statistics >10. In the *F*-statistic, the precision and magnitude of the effect of SNP on constipation were explored. *F*-statistics of individuals were 21–27. Using the IVW method, constipation was not causally related to BMI risk [OR = 1.010 (0.998, 1.022); *p* = 0.089] (Supplementary Table S3; Supplementary Figures S5A–C).

##### 3.1.2.2 Impact of constipation on WHR

We obtained 11 independent SNPs with genome-wide significance from GWAS. The SNPs used for the MR study were “strong” IVs with F-statistics >10. In the F-statistic, the precision and magnitude of the effect of SNP on WHR were analyzed. The IVW analysis exhibited no causal association between constipation and WHR risk (Supplementary Table S3; Supplementary Figures S8A–C).

##### 3.1.2.3 Impact of constipation on WC

There were 10 independent SNPs with genome-wide significance acquired from constipation GWAS. The SNPs used for the MR study were “strong” IVs whose *F*-statistics were > 10. In the *F*-statistic, the precision and magnitude of the effect of SNP on WC were examined. The IVW analysis did not show any causal correlation between constipation and WC risk (Supplementary Table S3; Supplementary Figures S7A–C).

##### 3.1.2.4 Impact of constipation on HC

This study acquired 10 independent SNPs with genome-wide significance from the GWAS. The SNPs utilized for the MR study were “strong” IVs whose *F*-statistics were > 10. In the *F*-statistic, the precision and magnitude of the effect of SNP on HC were explored. The IVW analysis did not exhibit any causal association between constipation and HC risk (Supplementary Table S3; Supplementary Figures S6A–C).

#### 3.1.3 Sensitivity and heterogeneity analyses

Supplementary Tables S2–S4 display more detailed sensitivity analysis results. Supplementary Figures S1B–S10B presents the leave-one-out plot. Supplementary Figures S1A–S10A exhibits the funnel plot. Some MR analyses revealed significant heterogeneity (*p* value<0.05). We used random-effects IVW to eliminate this heterogeneity as much as possible. The absence of pleiotropic effects was confirmed in all MR analyses (*p*_pleiotropy>0.05).

### 3.2 Meta-analysis

#### 3.2.1 Publication screening

A total of 2,228 potential publications were obtained from primary retrieval. Among them, 1,427 were removed by title and abstract reading. Full texts of the rest of the 481 publications were read. Finally, 14 publications were obtained for the final meta-analysis (Supplementary Figure S5).

#### 3.2.2 Publication features

A total of 14 publications were included in this meta-analysis, comprising 14 datasets (*n* = 43,488) and published between 1990 and 2024 (10, 11, 31–41). Supplementary Table S5 presents the main characteristics of all those 14 publications. There were 1,790 children and adolescents in the studies used for this meta-analysis.

The studies were carried out in different regions, including five in North America, one in Europe, one in Oceania, five in Asia, and two in South America. The constipation patient numbers were 6–5,781. Supplementary Table S5 exhibits the study quality score rated by the nine-star NOS or AHRQ system. Therefore, five publications (12, 33, 34, 37, 40) had seven points, seven (10, 11, 31, 32, 36, 38, 41) had eight points, and two (35, 39) had nine points. Following quality assessment criteria, all publications in this meta-analysis had moderate or high quality.

#### 3.2.3 Meta-analysis between overweight, obesity, and constipation

The Egger test was used to analyze the possible publication bias in our included studies. Meanwhile, a funnel plot was drawn to evaluate the publication bias risk, which revealed obvious publication bias in one study. Thus, the study performed by Olaru et al. (32) was excluded from our analysis. The rest studies had low bias risk (Supplementary Figure S11).

**Figure 4:**
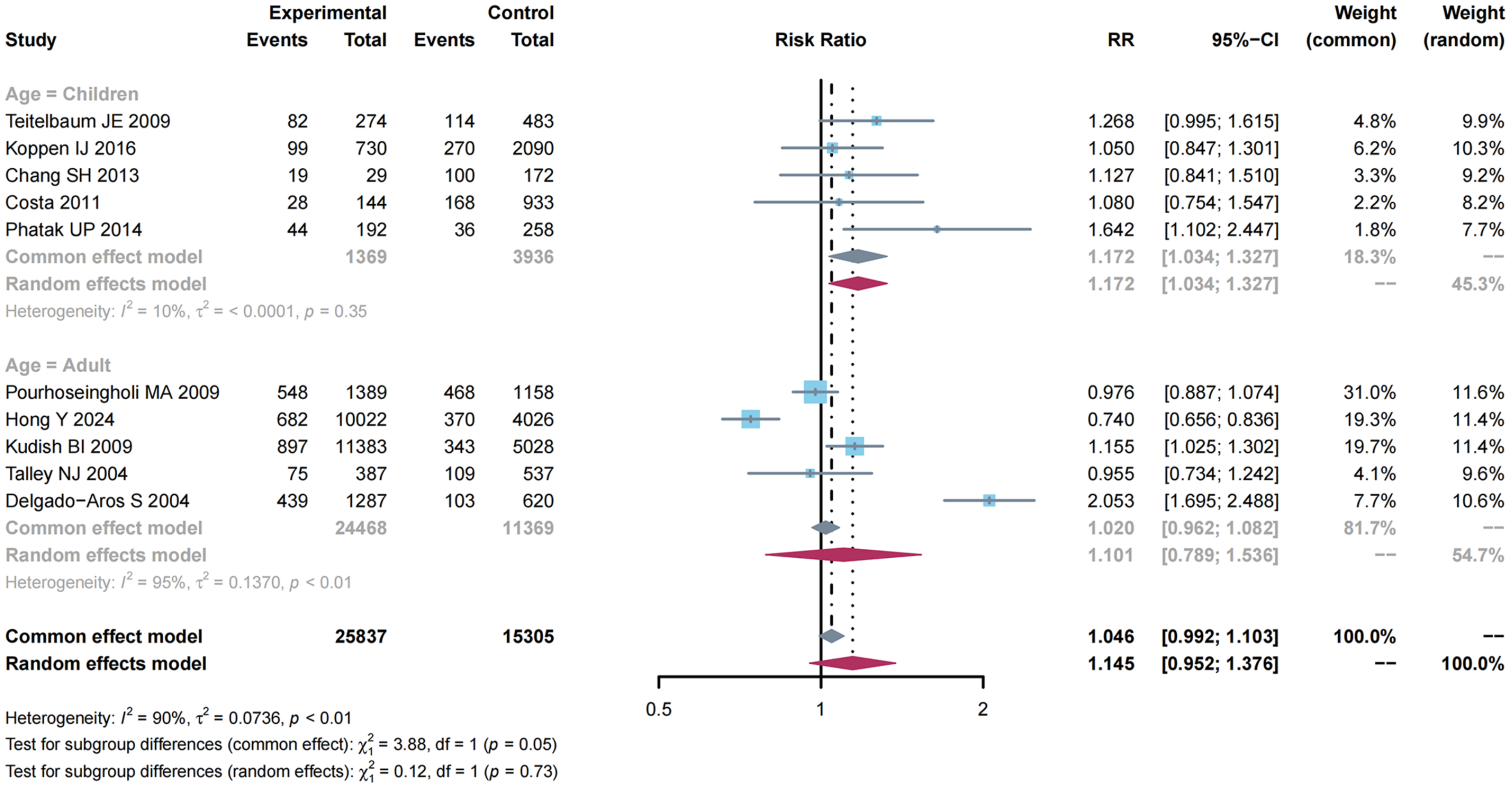
Subgroup analysis of age for the associations between overweight and obesity and the risk of constipation.

There were 10 studies (10–12, 31, 33, 35, 37–40) mentioning risk estimates regarding overweight and obesity and constipation risk. Due to the significant heterogeneity (*I*^2^ = 90%, *p* < 0.01; Figure 4), a random-effects model was adopted for this meta-analysis, and a higher constipation risk was detected (RR: 1.145; 95% CI: 0.952–1.376).

From Figure 4, subgroup analysis was employed to analyze factors related to constipation risk in children compared with adult groups. Significant heterogeneity was not detected (*I*^2^ = 10%, *p* = 0.35). Therefore, a common-effect model was used for meta-analysis. As a result, there was a higher constipation risk (RR: 1.172; 95% CI: 1.034–1.327). Nevertheless, due to the obvious heterogeneity (*I*^2^ = 95%, *p* < 0.01) among the adult group, a random-effects model was used for meta-analysis, and a higher constipation risk was detected (RR: 1.101; 95% CI: 0.789–1.536).

#### 3.2.4 Meta-analysis between overweight and constipation

Egger test was carried out to evaluate the possible publication bias of the included publications, revealing no obvious publication bias (*p* = 0.4497). Moreover, a funnel plot was drawn to evaluate the publication bias risk (Supplementary Figure 5), and no obvious publication bias was found.

There were eight studies (10, 11, 31, 33, 37–40) reporting estimates of overweight with constipation risk. An obvious heterogeneity was determined (*I*^2^ = 58%, *p* = 0.02; Supplementary Figure S12). Thus, a random-effects model was used for meta-analysis, exhibiting a decreased constipation risk (RR: 0.985; 95% CI: 0.884–1.097).

**Figure 5:**
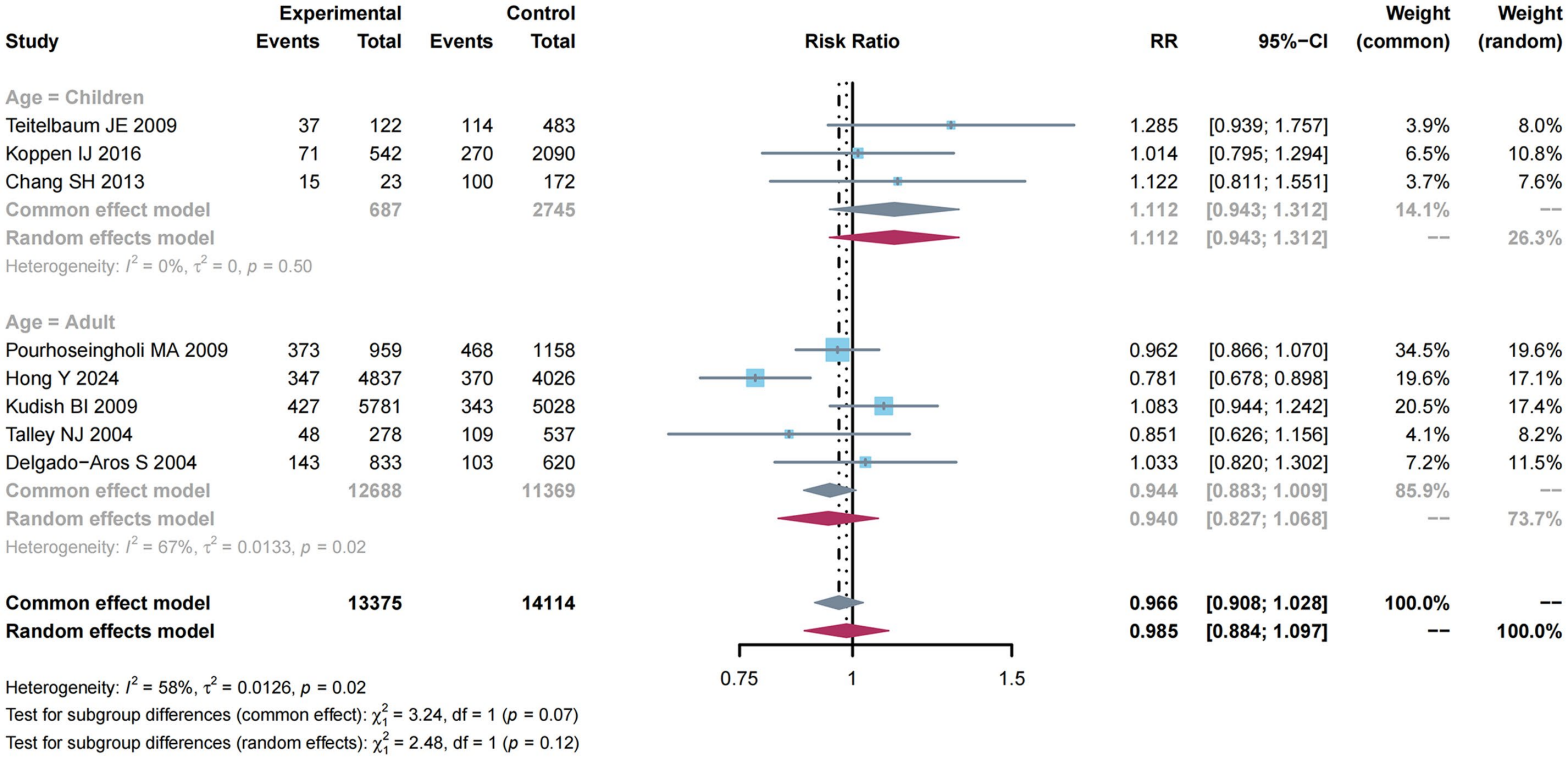
Subgroup analysis of age for the associations between overweight and the risk of constipation.

As shown in Figure 5, subgroup analysis was performed to analyze the factors related to constipation risk in Children compared with adult groups. Significant heterogeneity was not detected (*I*^2^ = 0%, *p* = 0.50), and thus, we utilized the common-effect model for meta-analysis and determined a higher constipation risk (RR: 1.112; 95% CI: 0.943–1.312). Nevertheless, owing to the obvious heterogeneity (*I*^2^ = 67%, *p* = 0.02) in the adult group, a random-effects model was used for meta-analysis, discovering a decreased constipation risk (RR: 0.940; 95% CI: 0.827–1.068).

#### 3.2.5 Meta-analysis between obesity and constipation

Egger test was employed to evaluate the possible publication bias across our included publications. Meanwhile, a funnel plot was utilized to evaluate the publication bias risk, suggesting obvious publication bias in one study. Thus, the study performed by Olaru et al. (32) was excluded from our analysis. The rest publications had a low risk of bias (Supplementary Figure S13).

There were 10 studies (10–12, 31, 33, 36–39, 41) reporting estimates of obesity with constipation risk. Due to the obvious heterogeneity (*I*^2^ = 88%, *p* < 0.01), this study utilized a random-effects model for meta-analysis and found a higher constipation risk (RR: 1.186; 95% CI: 0.989–1.420).

**Figure 6:**
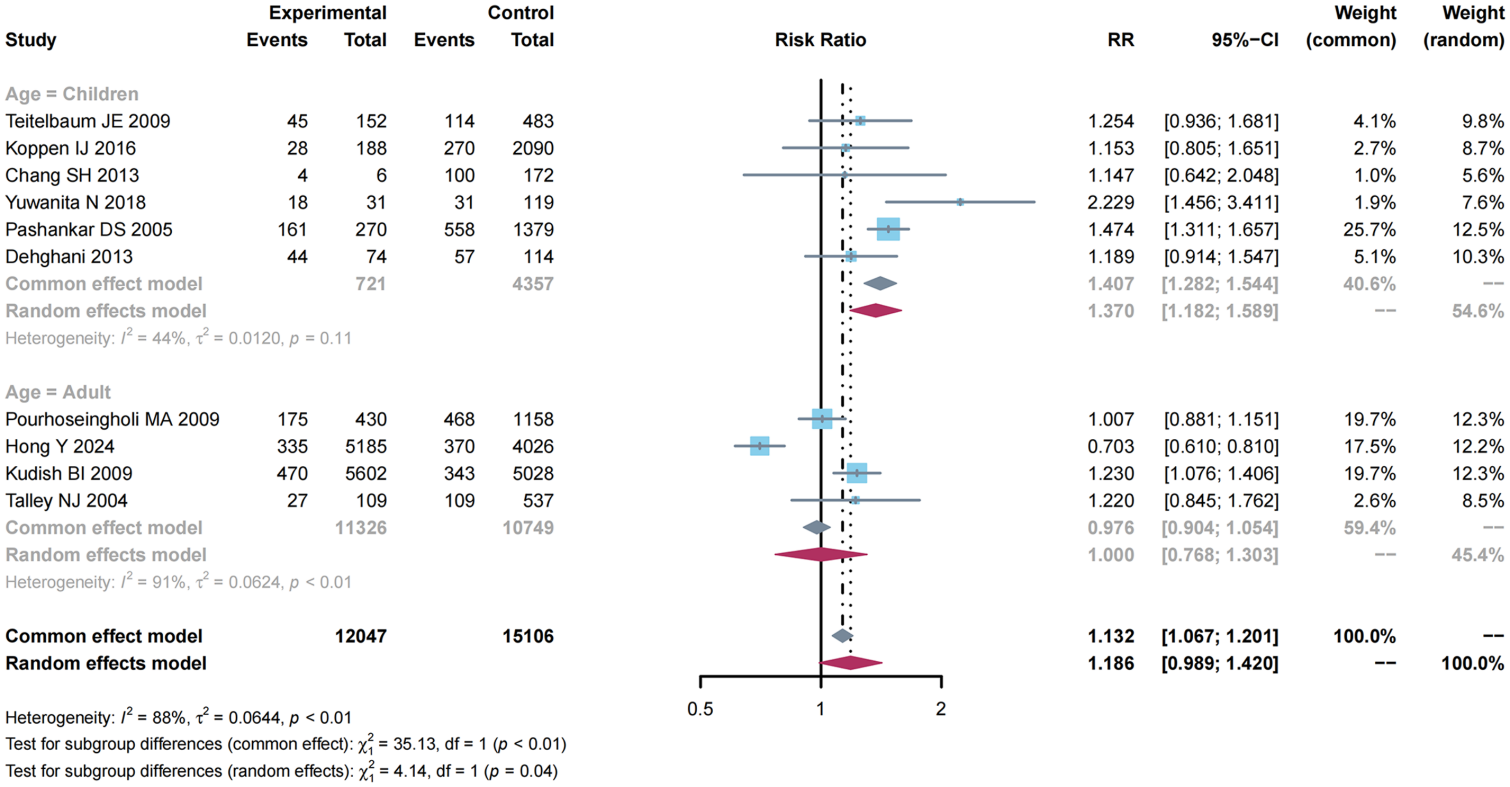
Subgroup analysis of age for the associations between obesity and the risk of constipation.

As presented in Figure 6, subgroup analysis was performed to examine factors related to constipation risk in Children compared with adult groups. Due to the absence of obvious heterogeneity (*I*^2^ = 44%, *p* = 0.11), the common-effect model was used for meta-analysis and found a higher constipation risk (RR: 1.407; 95% CI: 1.282–1.544). Nevertheless, considering the significant heterogeneity (*I*^2^ = 91%, *p* < 0.01) among the adult group, this study utilized a random-effects model, but did not reveal any impact of adults on constipation risk (RR: 1.000; 95% CI: 0.786–1.303).

## 4 Discussion

Based on the pooled GWAS data, this study comprehensively analyzed the causal association of obesity with constipation. Both MR study and meta-analysis were carried out in this study to investigate the association between obesity and constipation risk. The use of bidirectional MR contributes to identifying high-risk populations and potential genetic targets, offering data support for personalized medicine and precision treatment. In combined treatments for obesity and constipation, MR analysis can identify specific genetic susceptibilities, promoting individualized interventions based on genetic characteristics. This innovative approach, grounded in causal inference, provides novel perspectives and directions for the prevention and management of obesity and constipation.

As revealed by our results, BMI and WC showed causal associations with constipation. Various weight levels are associated with different health outcomes, which can be assessed by BMI. In this study, BMI was positively correlated with constipation. Constipation is more likely to occur with the increase in BMI. By contrast, BMI and constipation are non-linearly related, as found in the study by Xiang (7). In the study by Xiang (42), as BMI increases, constipation incidence initially decreases, while the risk rises significantly once BMI exceeds 28 kg/m^2^. WC is not associated with constipation in our study. To the best of our knowledge, only a few studies have examined the relationship between WC and constipation. Yurtdas (43) identified WC as a possible risk factor for constipation in women, which is consistent with our results, and higher WC was associated with a higher risk of constipation. We analyzed the causal effects of WHR and HC on constipation, finding no causal association. Constipation has been linked to overweight/obesity in many studies, but the results are inconsistent. Pawłowska revealed no significant difference in body weight/BMI between children with constipation and the control group (44). In addition, other studies showed that constipation was linked to underweight (45–47). The BMI and colonic transit time were negatively correlated in a study of 354 constipated patients (48).

To demonstrate the results of MR, we conducted a meta-analysis involving 14 observational studies (with 43,488 subjects), including six case–control, six cross-sectional, and two cohort studies. Obesity or overweight was found to be significantly related to constipation (RR: 1.145; 95% CI: 0.952–1.376). In the subgroup analysis, we divided the incidence of constipation in overweight or obese people into children and adults. It was found that overweight in adults would lower the risk of constipation, while obesity in adults exerted little effect on constipation. Being overweight or obese in children increases the risk of constipation. Several previous studies have reported that overweight/obesity is one of the risk factors for constipation in children, while this is not entirely consistent with some current research findings (49). According to Pashankar et al. (36), children who were constipated had a greater prevalence of overweight/obesity. Compared with a healthy control group, Dehghani et al. (41) found that children with FC endured a significantly higher rate of obesity. It was indicated in another study that children with constipation are more likely to be overweight than those in the control group (50). In contrast, some studies found that children with chronic constipation were more likely to be underweight and to grow at a slower rate (45, 46, 51). In addition, early diagnosis and treatment of constipation could facilitate the growth of a child.

The study by Moayyedi (52) proposed that the increased risk of constipation in obese people might be directly related to their food intake off and that the excessive food intake causes the stomach to expand rapidly, transporting food to the small intestine. The volume increases, resulting in an increased osmotic load and subsequent delivery of more to the colon stool, and stool consistency increases, which leads to constipation. Direct endocrine effects of adipokine may also affect the movement of the gastrointestinal tract. Rajindrajith et al. (53) also reported that rapid gastric emptying and intestinal or colon rotation in obese children reduced transit time and altered secretory response might partly explain the relationship between constipation and obesity. In addition, overweight/obese children are more often associated with unhealthy drinking, eating habits, and lifestyle, as well as excessive intake of high-fat foods. In addition, too little high-fiber food intake and lack of exercise have a certain effect on the occurrence of constipation. Another possible mechanism is probably related to brain–gut neuropeptides, among which, neuropeptides including ghrelin, leptin, glucagon-like peptide-1, and cholecystokinin are vital for satiety, hunger, and gastrointestinal motility. Gastrointestinal neuropeptides (like ghrelin) are demonstrated to promote small intestinal and colonic transit and exert potent prokinetic function. Ghrelin level is reported to increase in normal-weighted individuals when compared with obese people (54).

Psychosocial diseases have been suggested to exert critical effects on the pathophysiology of obesity and constipation. In some studies, obese children usually develop psychosocial disorders, including anxiety, depression, or lack of self-esteem (54, 55). Such disorders are probably associated with early-life alterations in gut microbiota, like compositional alterations, therefore indicating the occurrence and maintenance of constipation (56, 57). Additionally, it is a critical factor for energy metabolism and obesity occurrence (58–60). Devkota et al. reported that dietary fat dramatically reorganized intestinal microbiota in animal models, causing ecological diseases and disturbing immune homeostasis (61). Similarly, obesity is related to microbial alterations, decreased bacterial diversity, and changed metabolic pathways (58). Therefore, overweight/obesity may lead to a higher constipation risk among children through several mechanisms. Our results are consistent with the results of Eslick’s (62) systematic review that obesity is not associated with the risk of adult constipation. In our study, obesity was considered to cause constipation, but constipation did not cause obesity. Consequently, more investigations are needed to explore the potential mechanisms and shed more light on their interactions in the pathogenesis of both diseases.

## 5 Strengths and limitations

There are some strengths in this study. First, this study mainly concentrated on population-based studies on obesity with a large study population (*n* = 43,488), providing complete and creditable findings in comparison with prior meta-analysis results. Second, studies were classified based on age and obesity degree; in addition, factors were adjusted, and their influence on the association of exposure with outcome was estimated. Third, this is the first MR analysis that evaluates the causal association of obesity with constipation. Moreover, the study design was in strict accordance with the MR assumptions (63), avoiding the impacts of possible confounders and reverse causality. This revealed an independent association of obesity with constipation. Fourthly, obesity-related and constipation-related SNPs were obtained from the currently most complete GWAS, contributing to explaining the genetic association of obesity with constipation and providing accurate estimates. Based on our knowledge, this is the first study examining the reciprocal causal association of obesity with constipation by MR methods. In addition, the genetic summary data in obese subjects were obtained from the Cochrane Library, Web of Science, PubMed, and Embase databases from inception to 1 February 2024, enhancing the statistical power for evaluating the causality.

Nevertheless, there are certain limitations in this study. At first, due to the restricted number of publications, the subjects enrolled for meta-analysis were mostly Asians and Caucasians, while summary data for the MR study were obtained from subjects of European descent. Consequently, more research is warranted to generalize the results to additional populations. In addition, because of the restricted source of IVs regarding obesity, we did not stratify the study population by confounders such as sex, drug use, and disease course, which inevitably induced possible bias resulting from these confounders. However, eliminating all types of confounding factors can bring a great challenge. In the future, we will use multiple omics or wet experiments to reveal the interaction between obesity and constipation.

## 6 Conclusion

Collectively, obese people are associated with an increased risk of constipation, and genetically predisposed obesity shows a causal association with a higher constipation risk. However, constipation does not exhibit any causal association with a higher obesity risk. More studies are needed to explore the contributing mechanism of obesity to constipation. In addition, our study suggests that we can develop interventions for constipation from the perspective of obesity. In addition, some people with chronic constipation may be able to relieve their constipation by controlling their weight.
