# Supplementary figures and images for: A causal association between obesity and constipation: a two-sample bidirectional Mendelian randomization study and meta-analysis
Source: Front Nutr. 2024 Nov 11;11:1430280. doi: 10.3389/fnut.2024.1430280 (PMC11586183; doi:10.3389/fnut.2024.1430280)

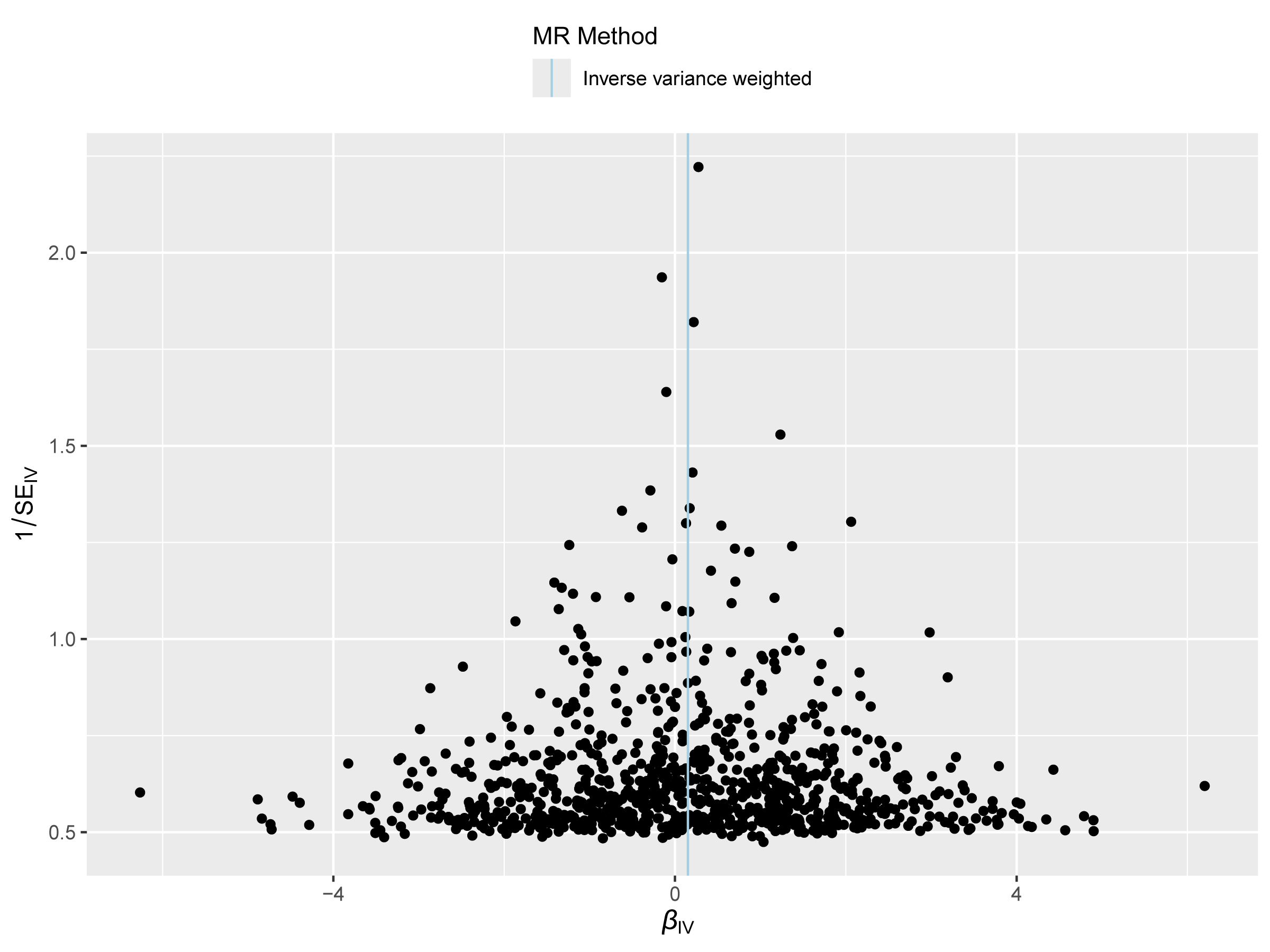

Supplement: Supplementary file 2 [file Data_Sheet_1.zip › supplementary figures/Figure S1 A.tif]

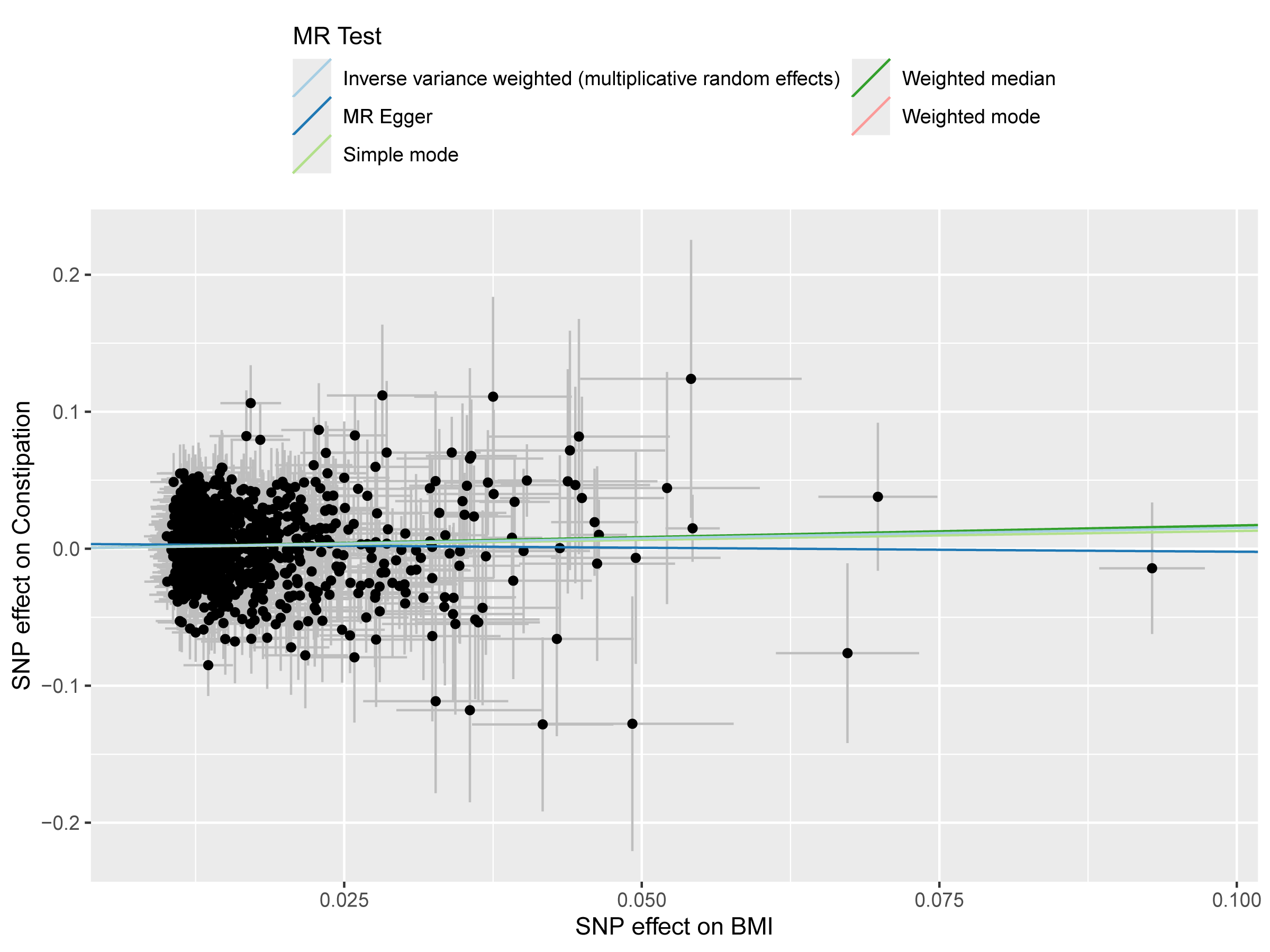

Supplement: Supplementary file 2 [file Data_Sheet_1.zip › supplementary figures/Figure S1 C.tif]

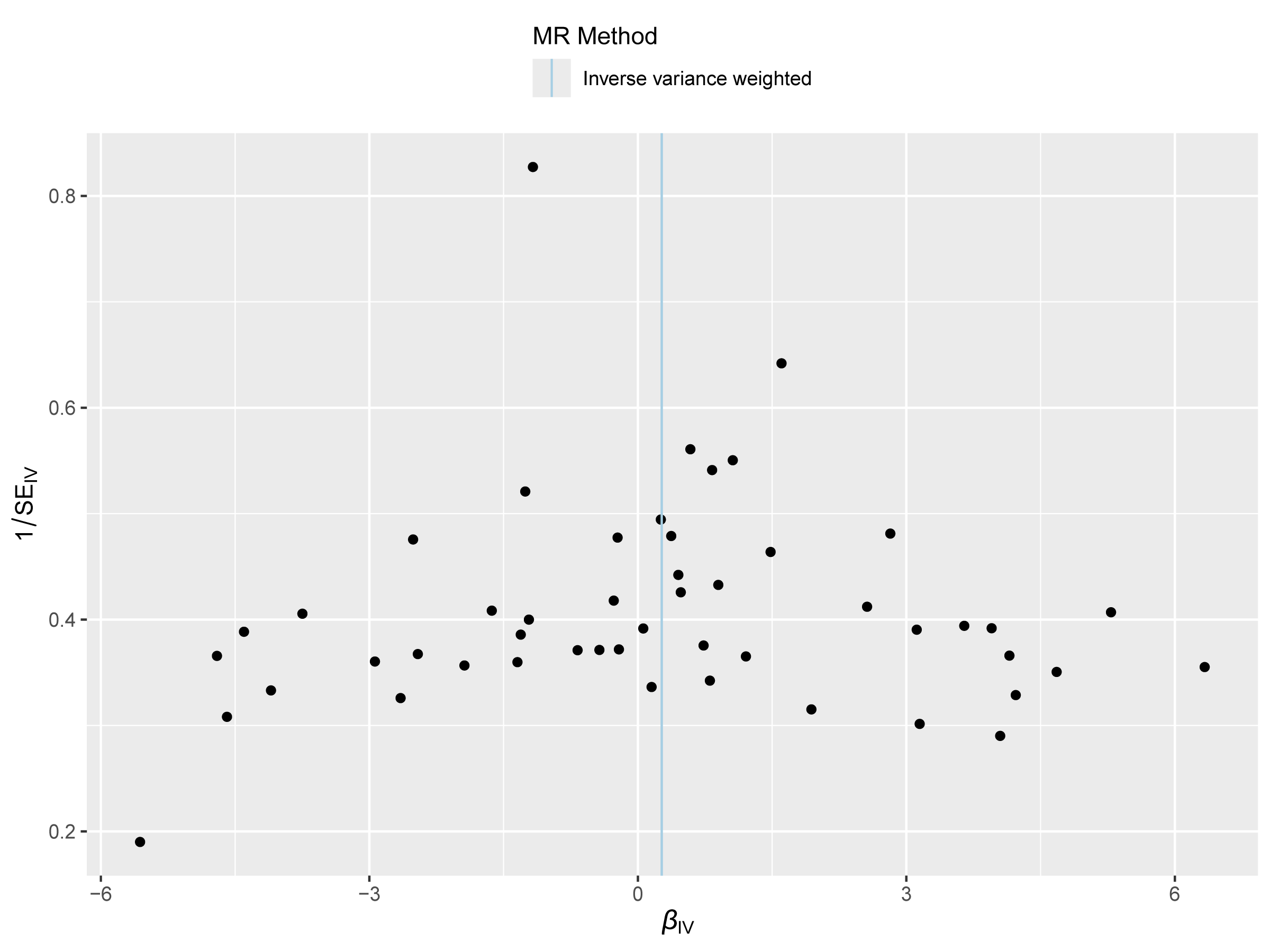

Supplement: Supplementary file 2 [file Data_Sheet_1.zip › supplementary figures/Figure S10 A.tif]

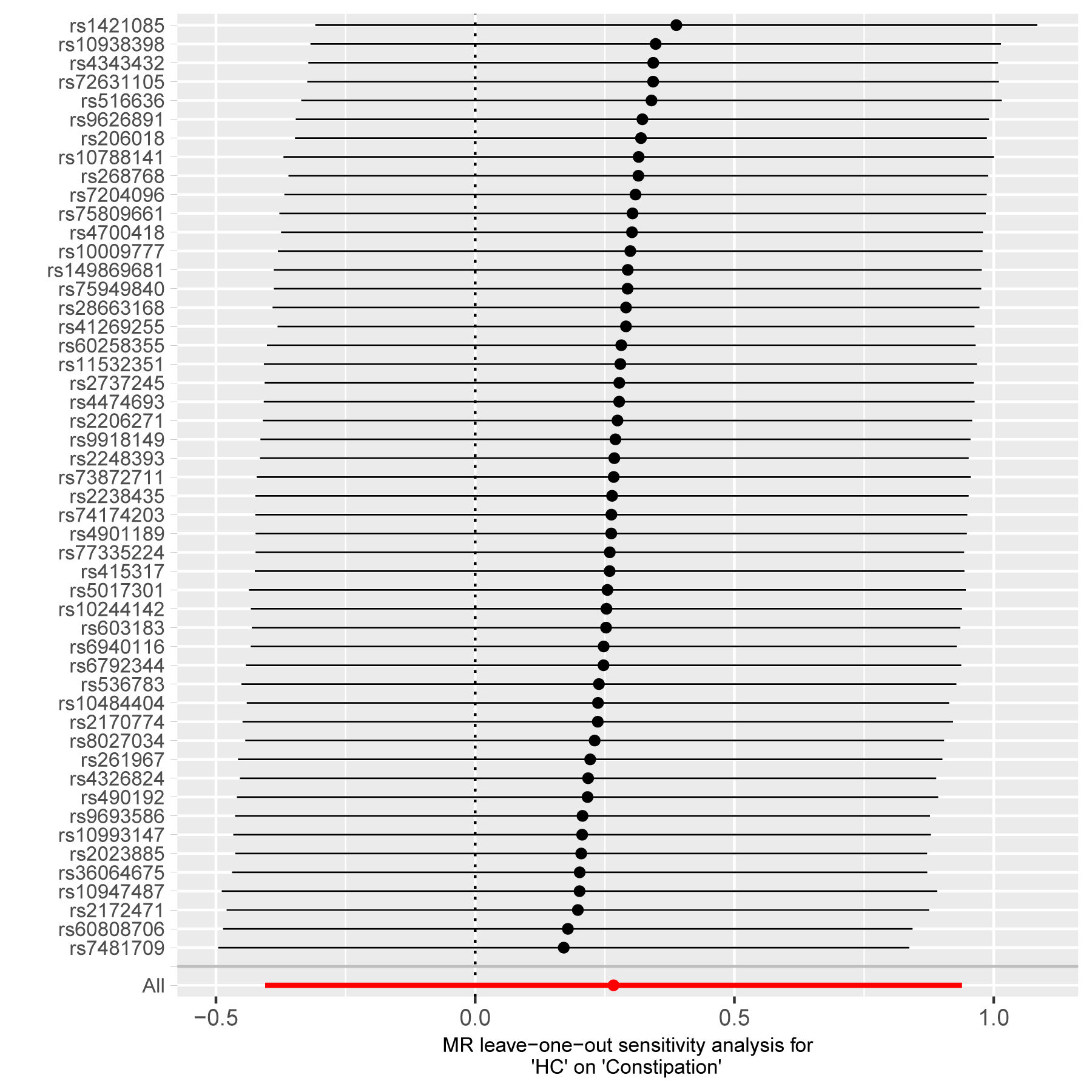

Supplement: Supplementary file 2 [file Data_Sheet_1.zip › supplementary figures/Figure S10 B.tif]

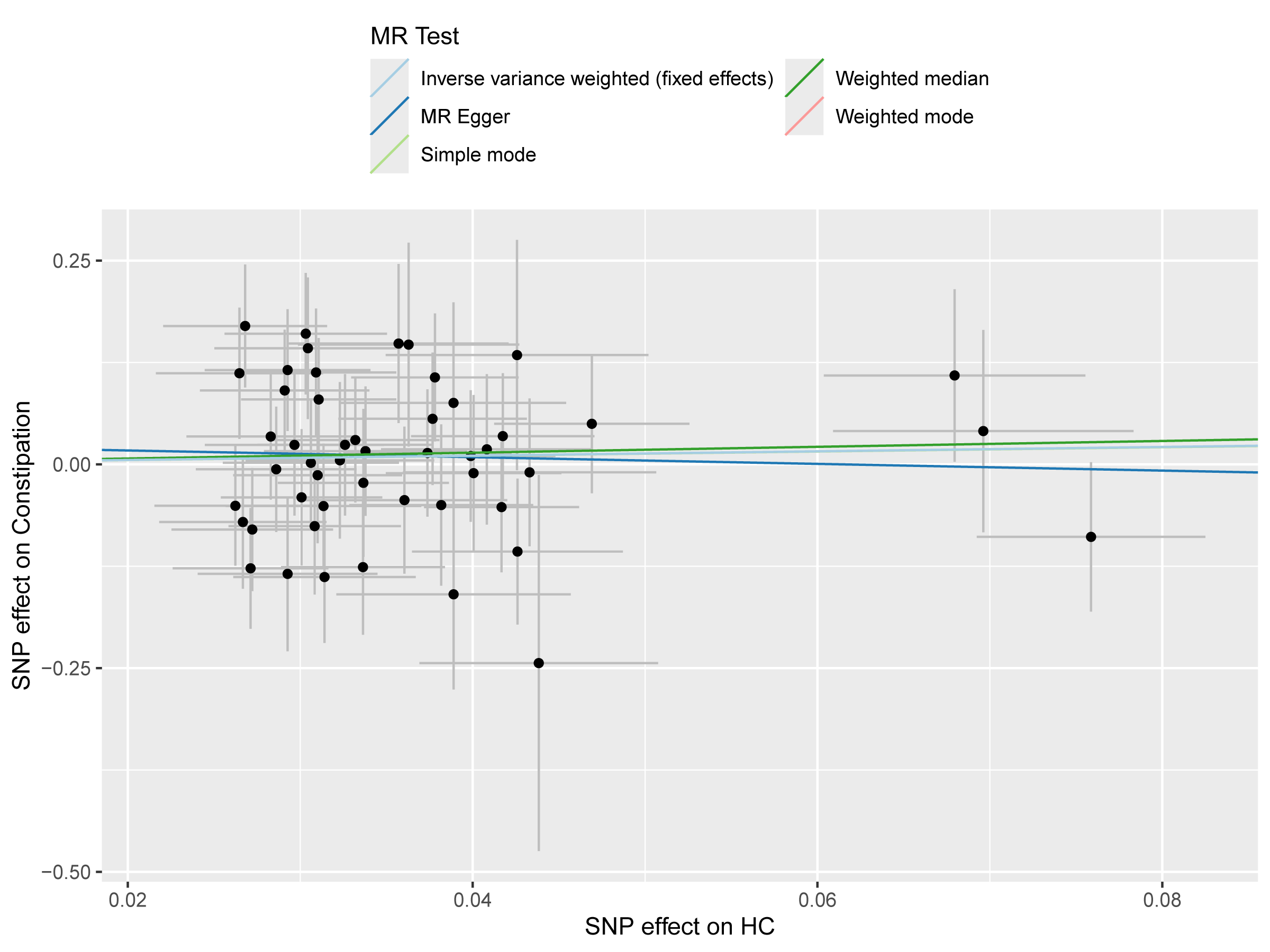

Supplement: Supplementary file 2 [file Data_Sheet_1.zip › supplementary figures/Figure S10 C.tif]

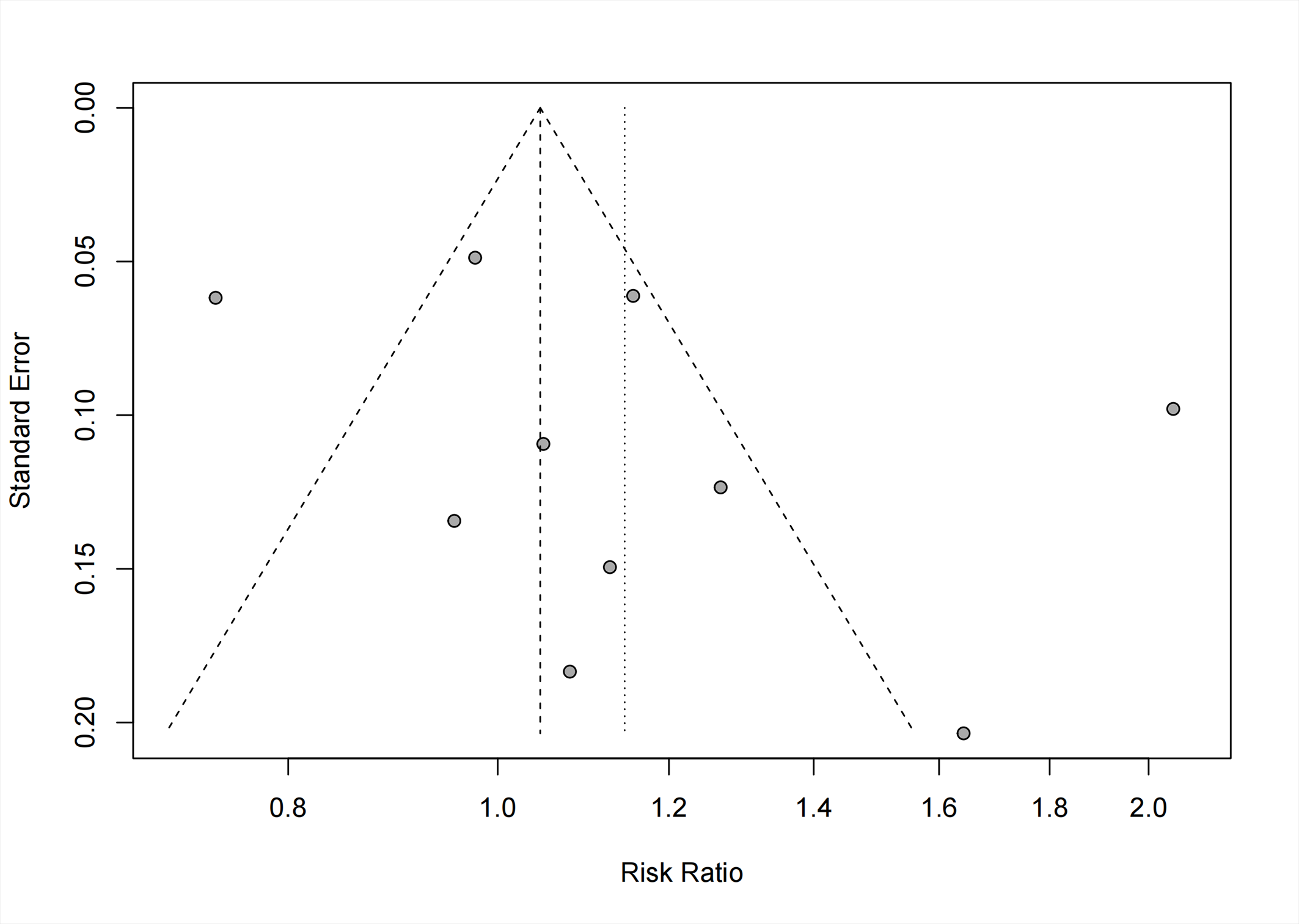

Supplement: Supplementary file 2 [file Data_Sheet_1.zip › supplementary figures/Figure S11.tif]

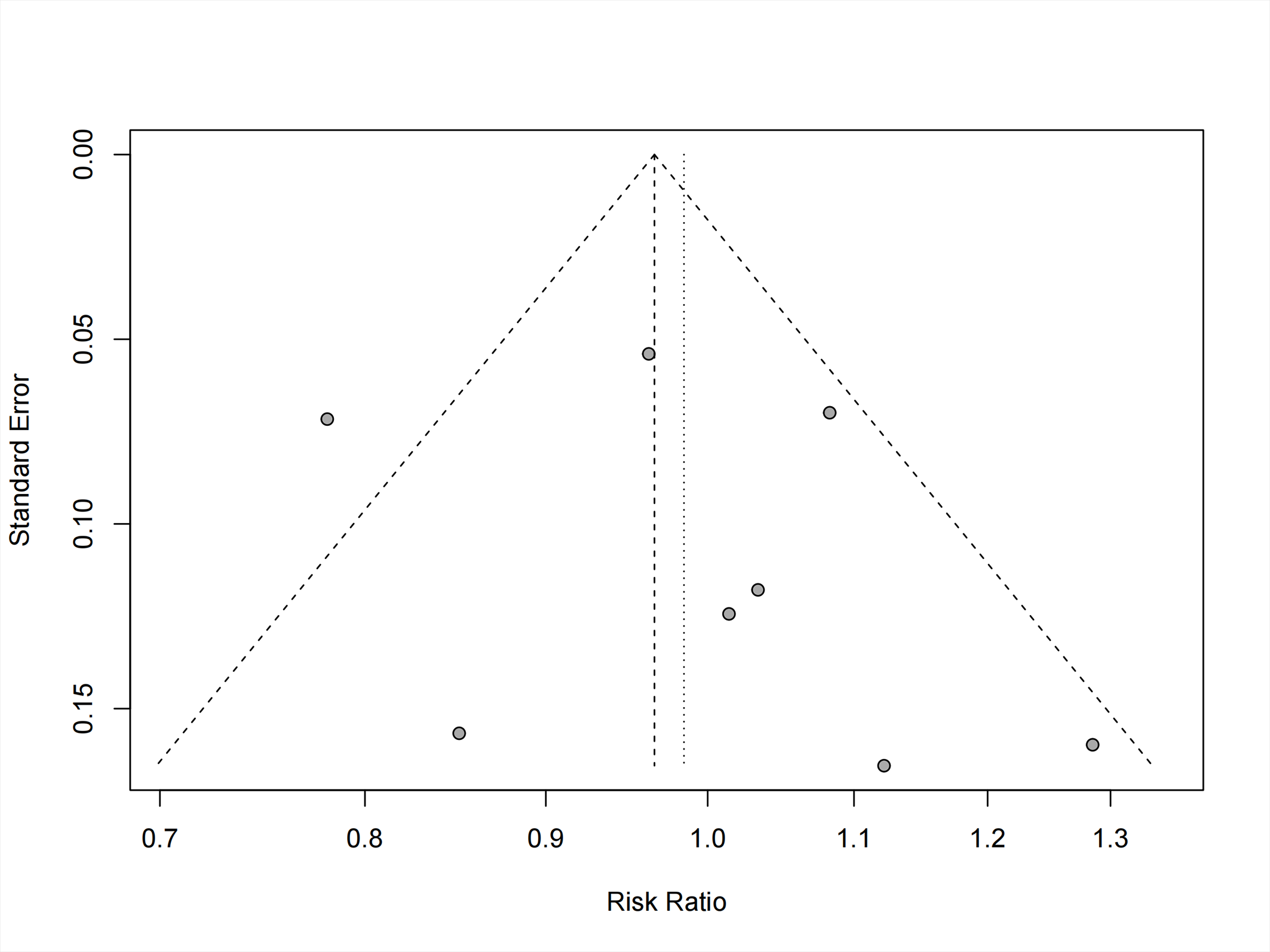

Supplement: Supplementary file 2 [file Data_Sheet_1.zip › supplementary figures/Figure S12.tif]

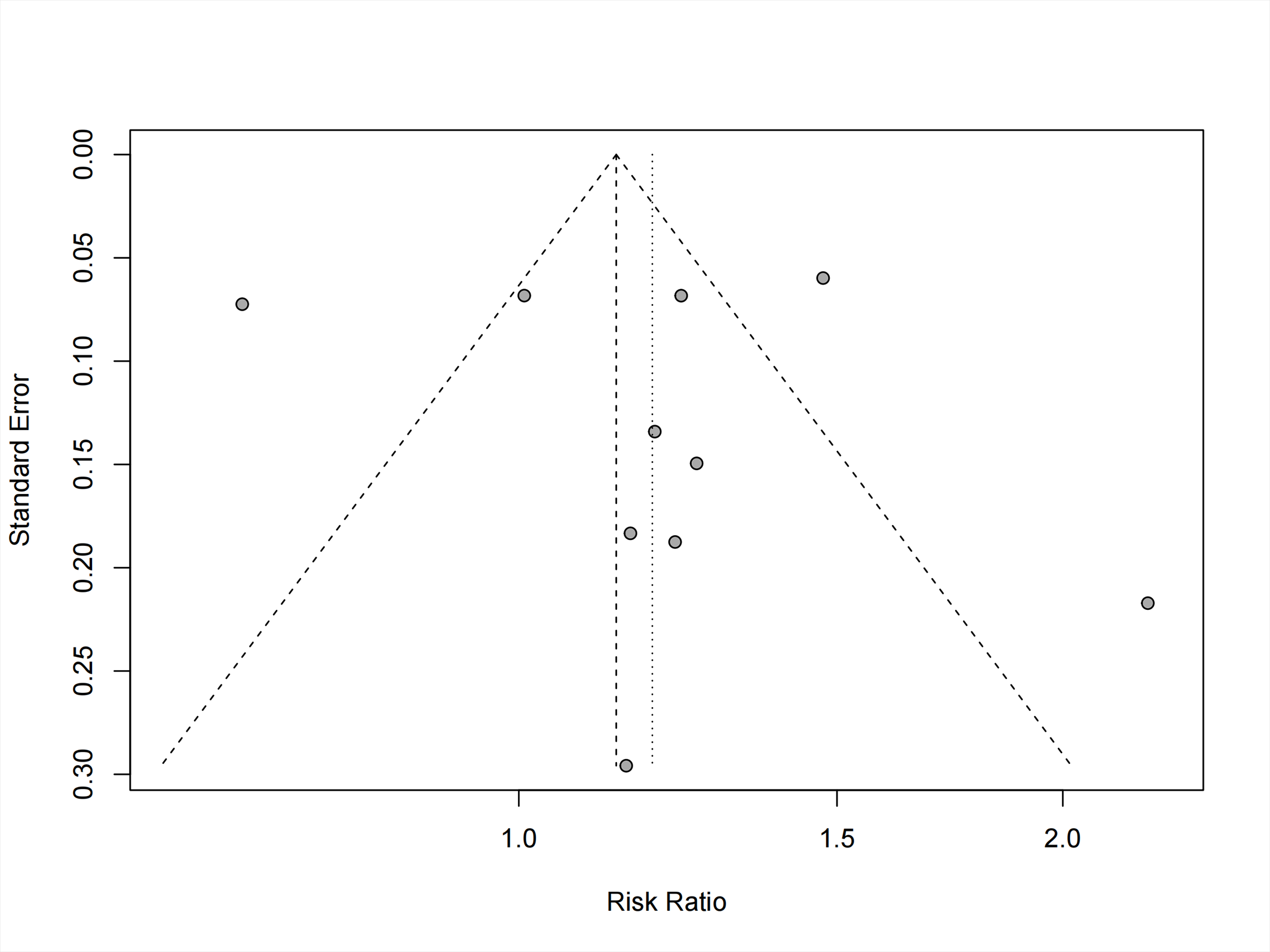

Supplement: Supplementary file 2 [file Data_Sheet_1.zip › supplementary figures/Figure S13.tif]

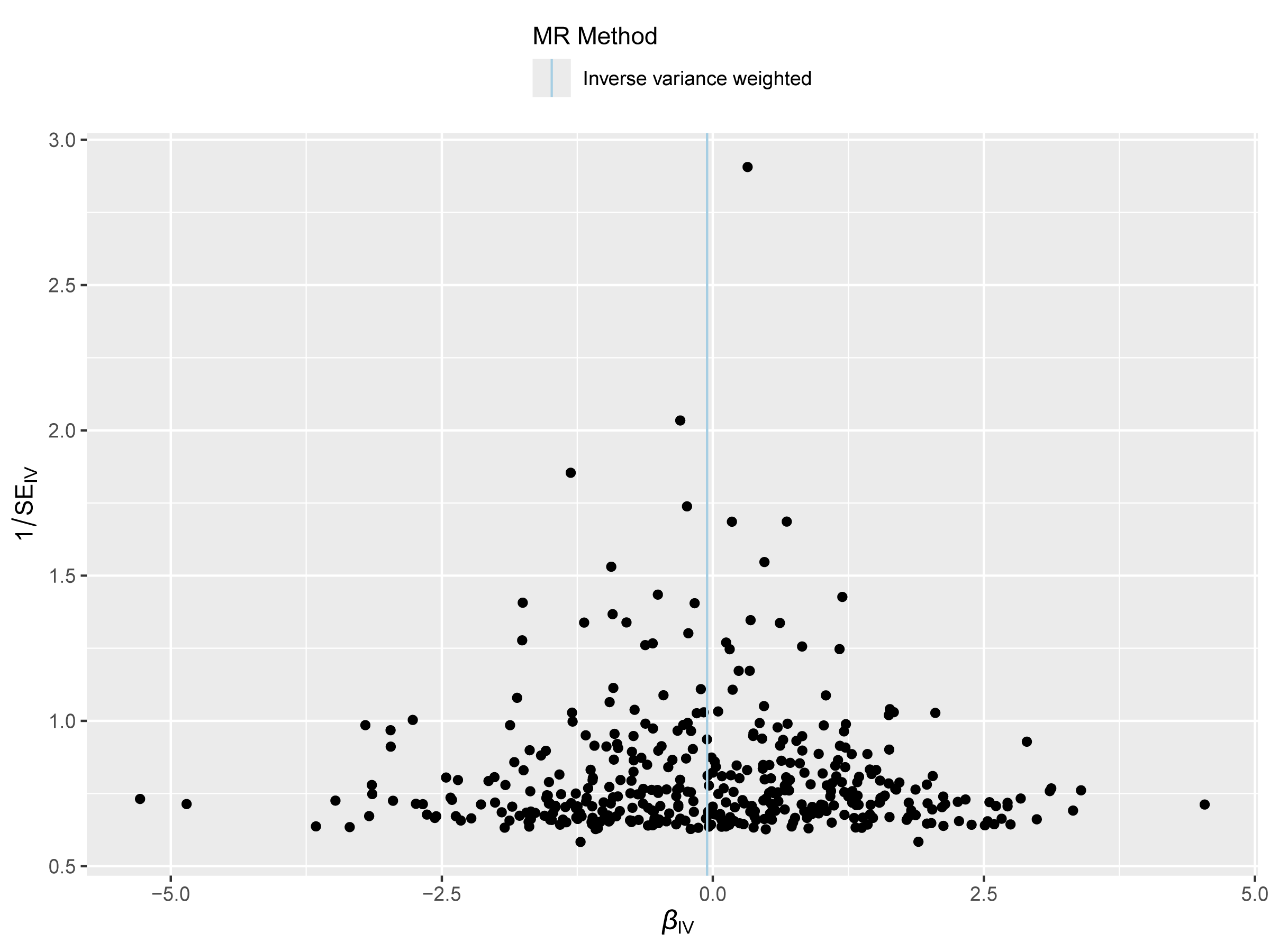

Supplement: Supplementary file 2 [file Data_Sheet_1.zip › supplementary figures/Figure S2 A.tif]

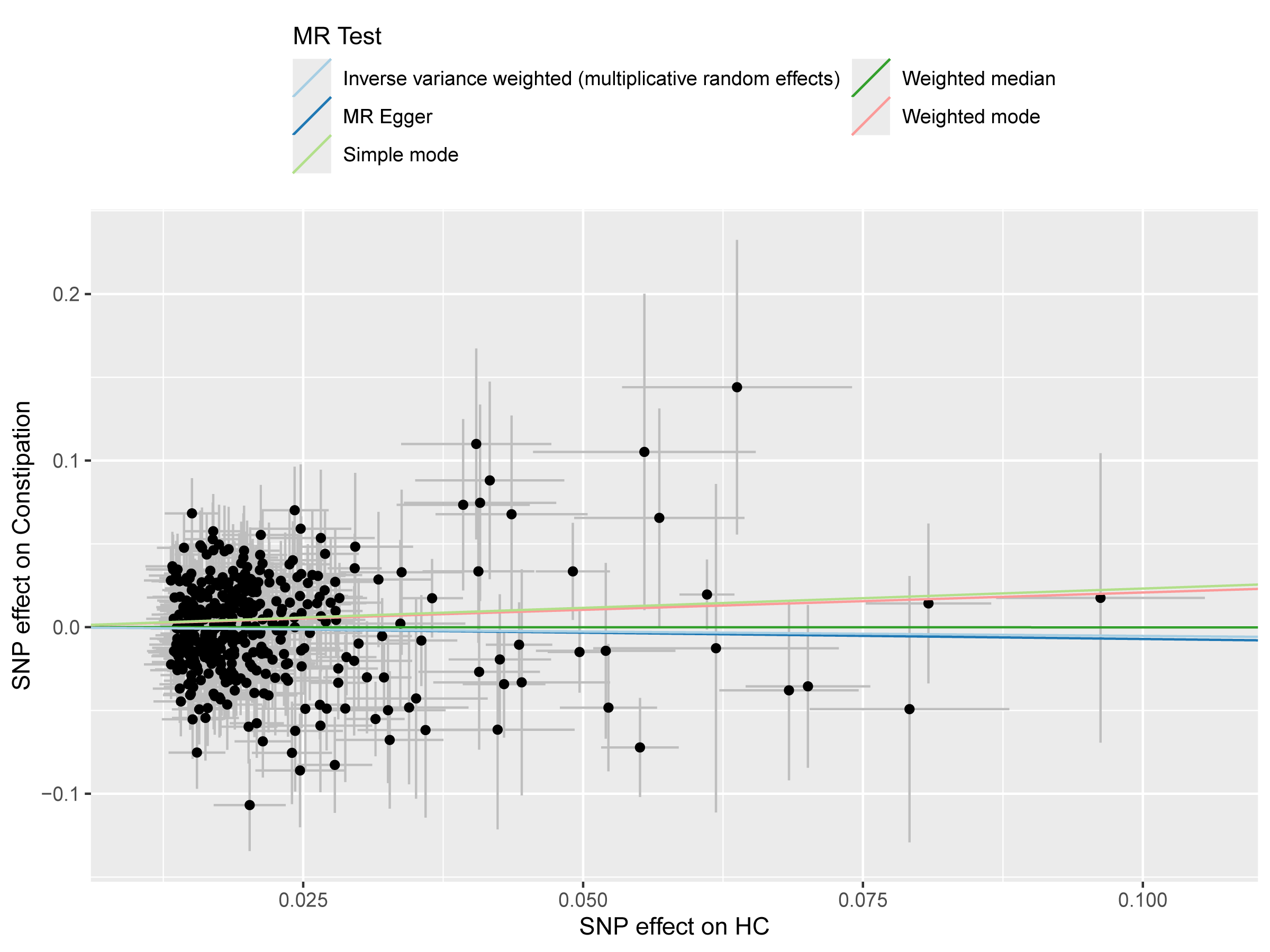

Supplement: Supplementary file 2 [file Data_Sheet_1.zip › supplementary figures/Figure S2 C.tif]

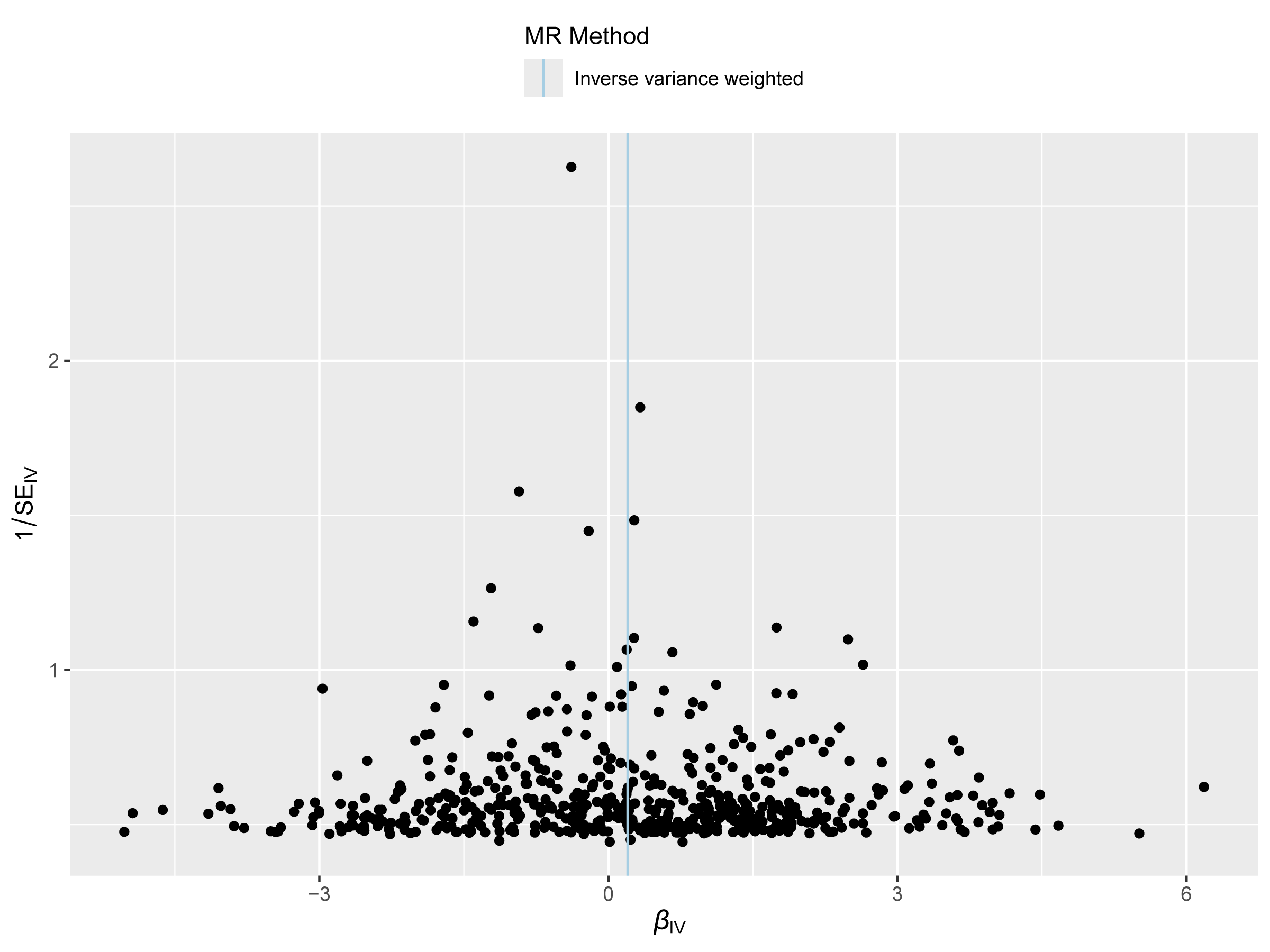

Supplement: Supplementary file 2 [file Data_Sheet_1.zip › supplementary figures/Figure S3 A.tif]

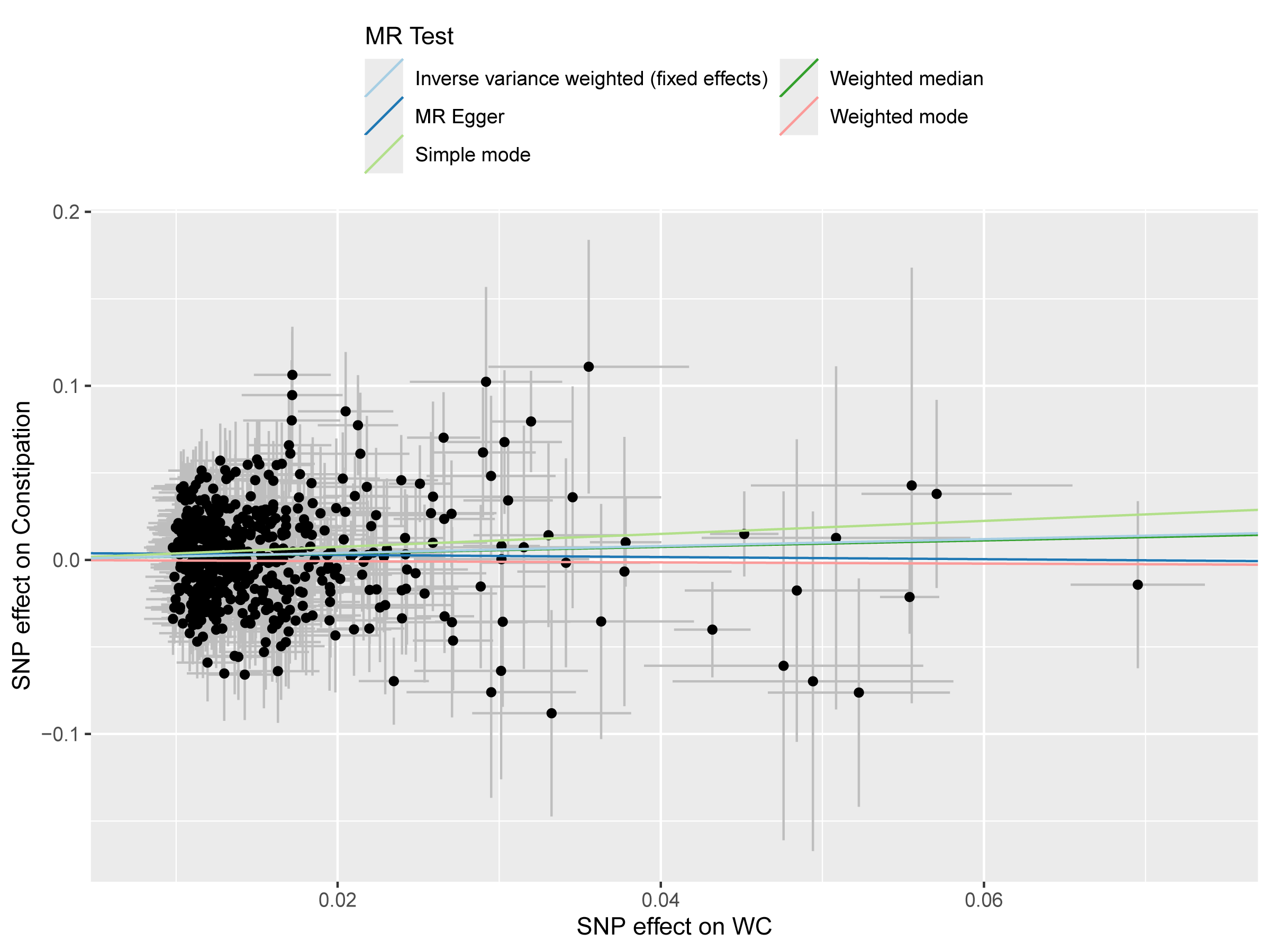

Supplement: Supplementary file 2 [file Data_Sheet_1.zip › supplementary figures/Figure S3 C.tif]

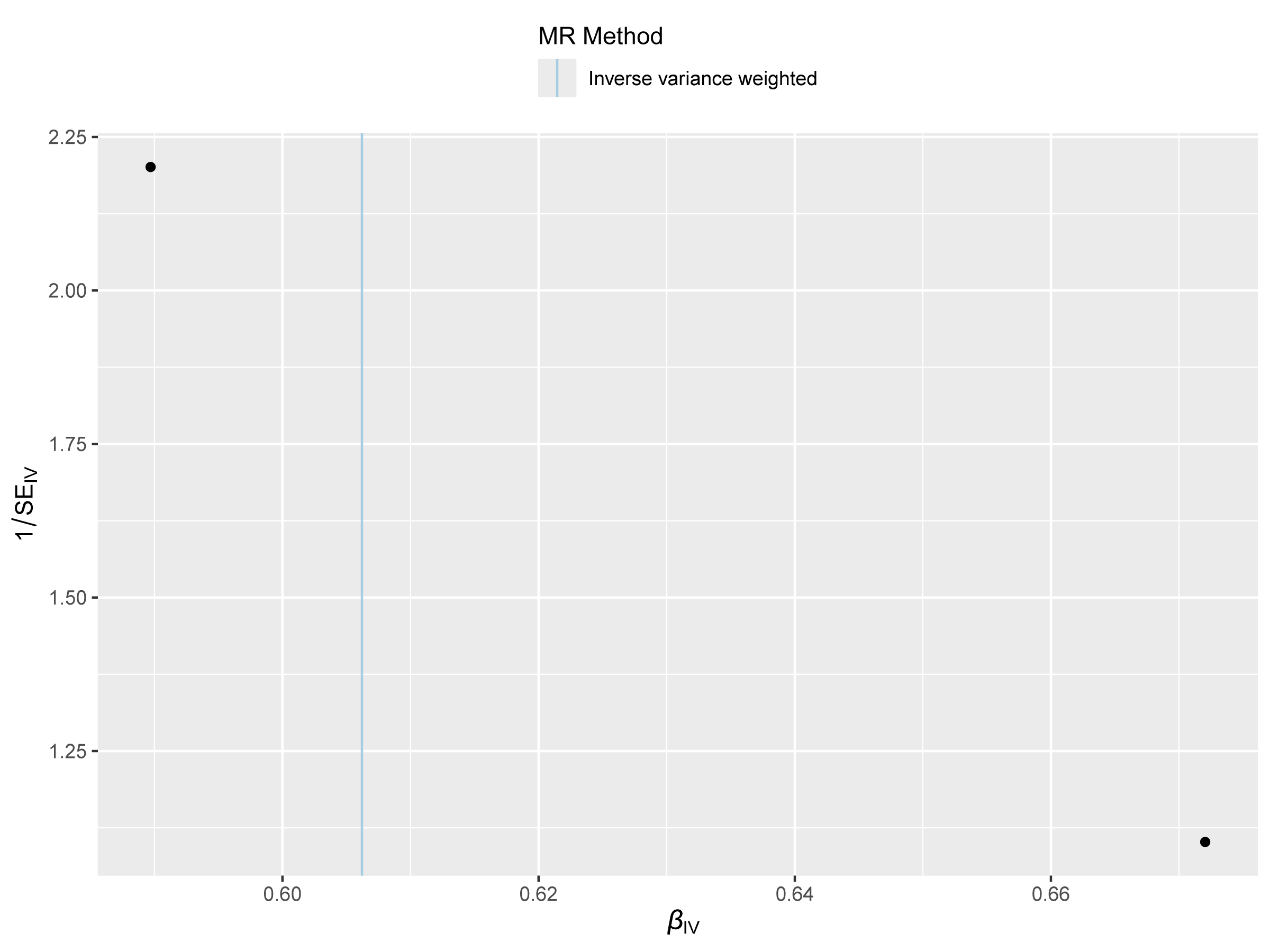

Supplement: Supplementary file 2 [file Data_Sheet_1.zip › supplementary figures/Figure S4 A.tif]

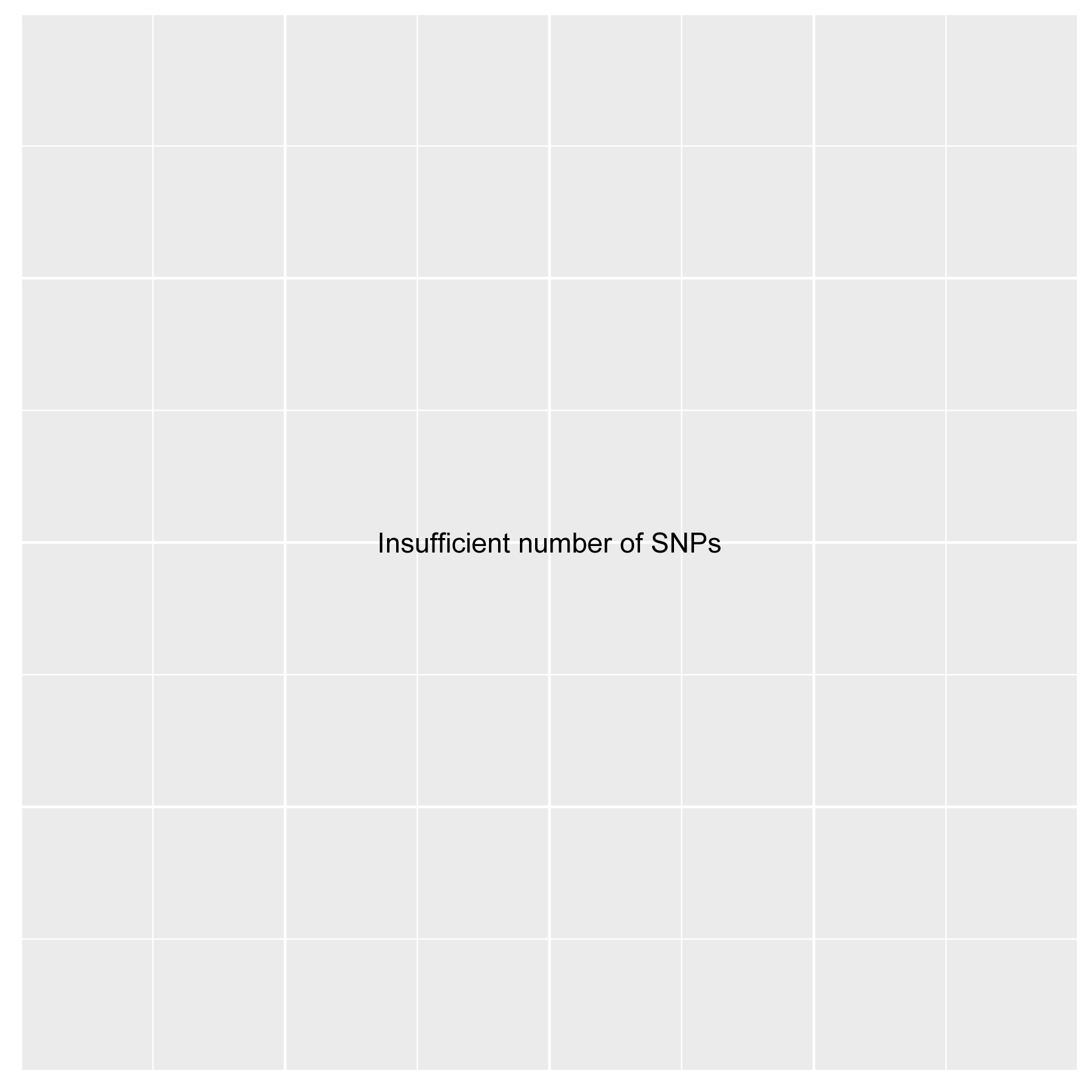

Supplement: Supplementary file 2 [file Data_Sheet_1.zip › supplementary figures/Figure S4 B.tif]

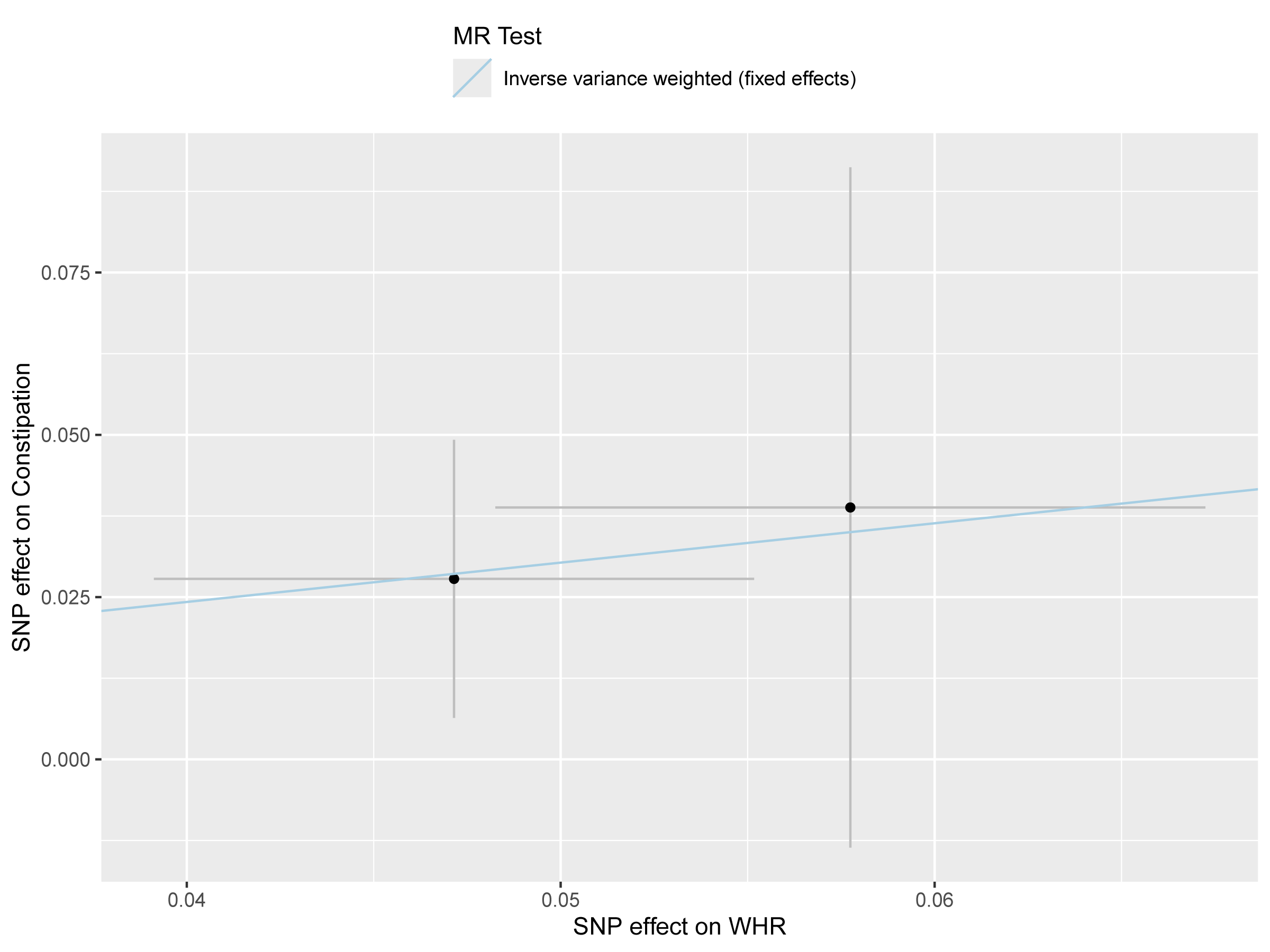

Supplement: Supplementary file 2 [file Data_Sheet_1.zip › supplementary figures/Figure S4 C.tif]

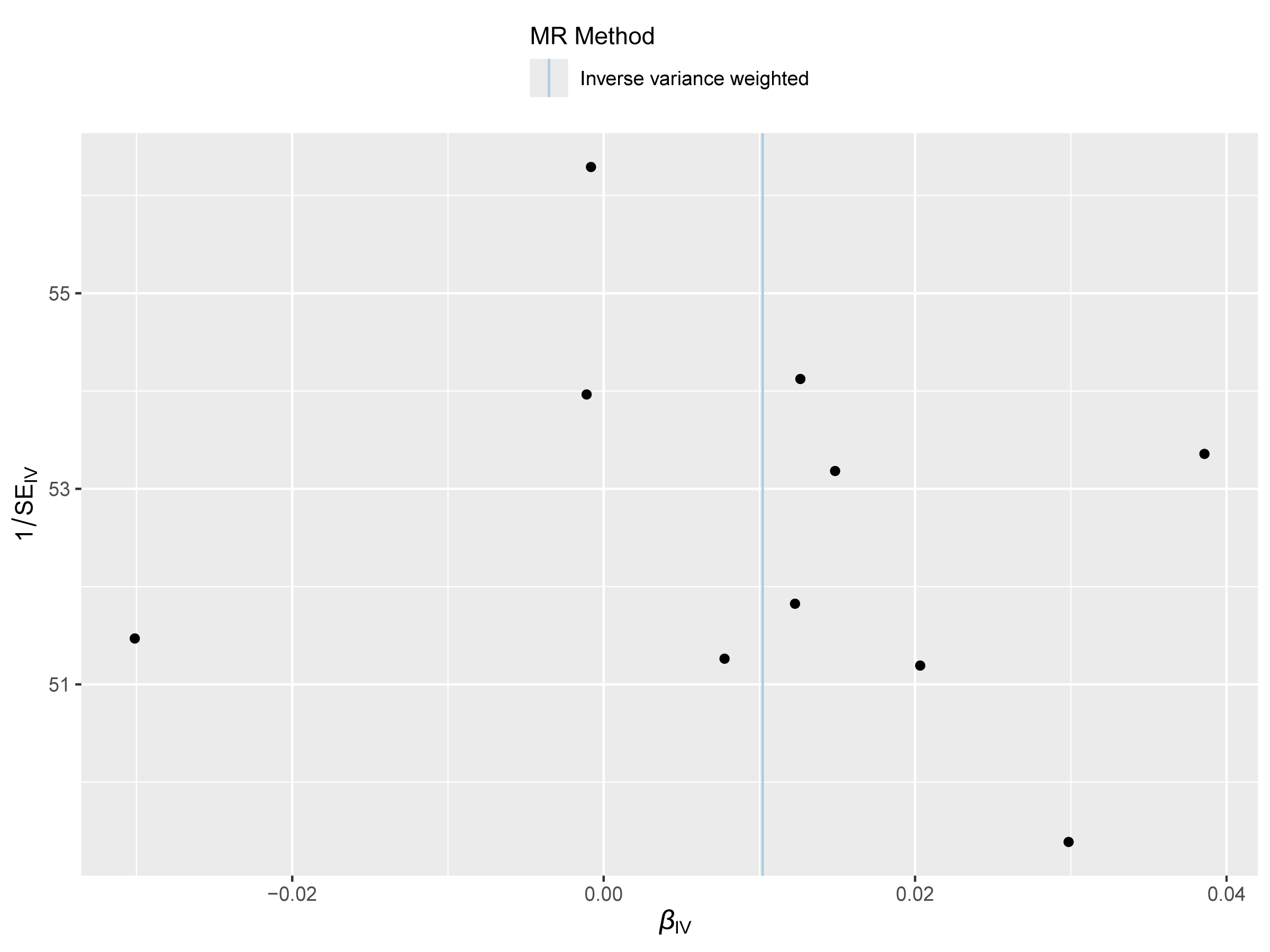

Supplement: Supplementary file 2 [file Data_Sheet_1.zip › supplementary figures/Figure S5 A.tif]

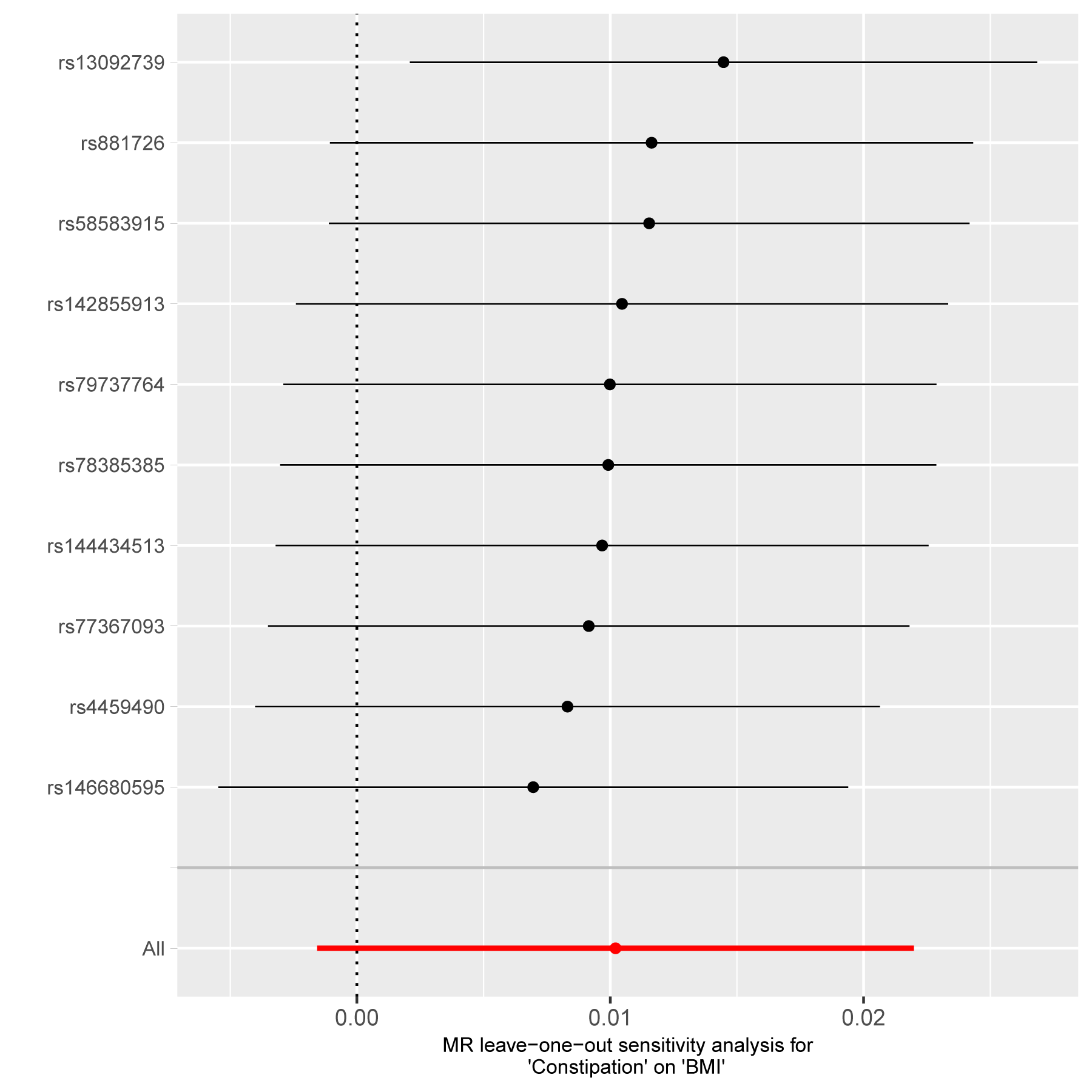

Supplement: Supplementary file 2 [file Data_Sheet_1.zip › supplementary figures/Figure S5 B.tif]

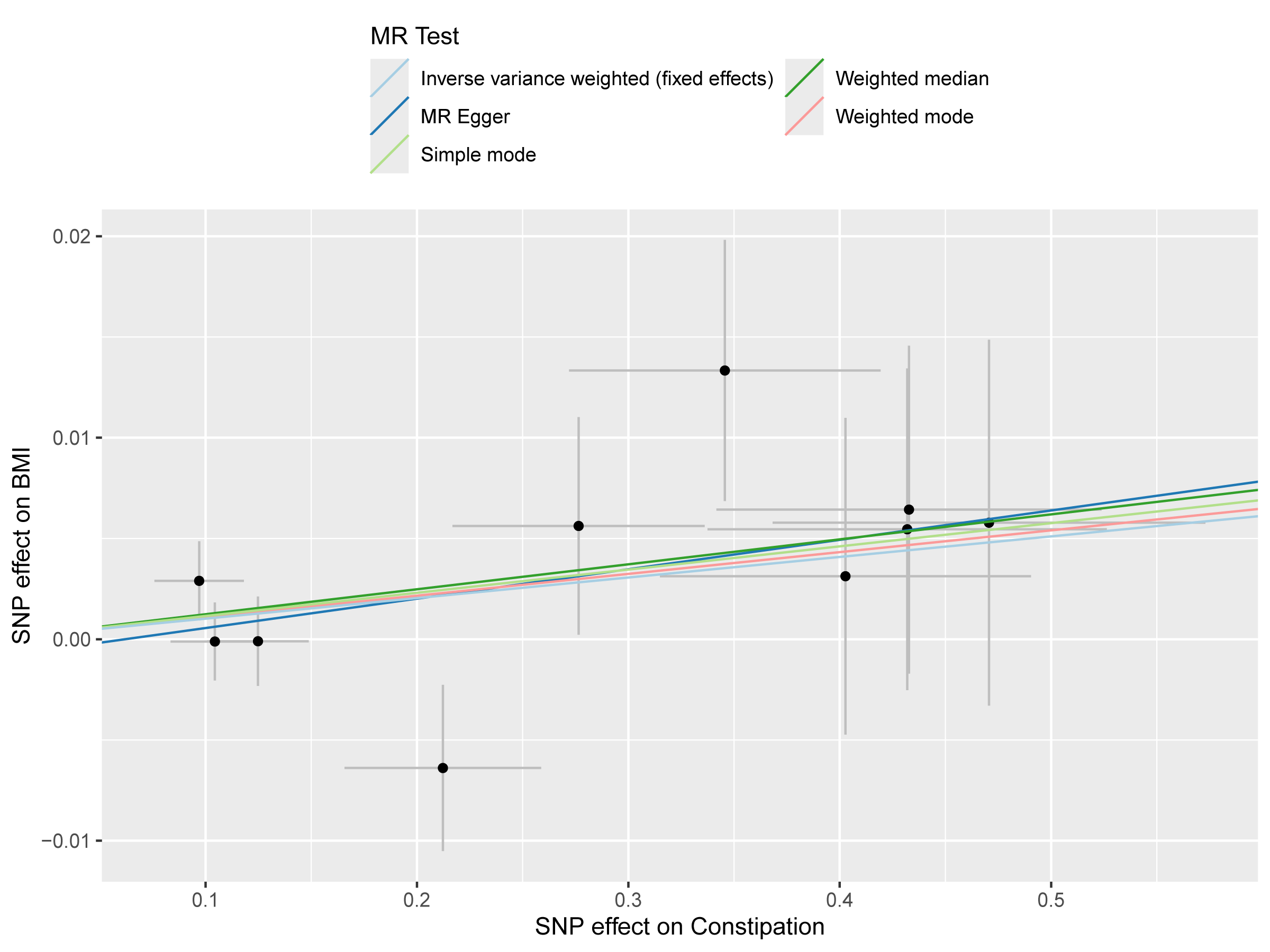

Supplement: Supplementary file 2 [file Data_Sheet_1.zip › supplementary figures/Figure S5 C.tif]

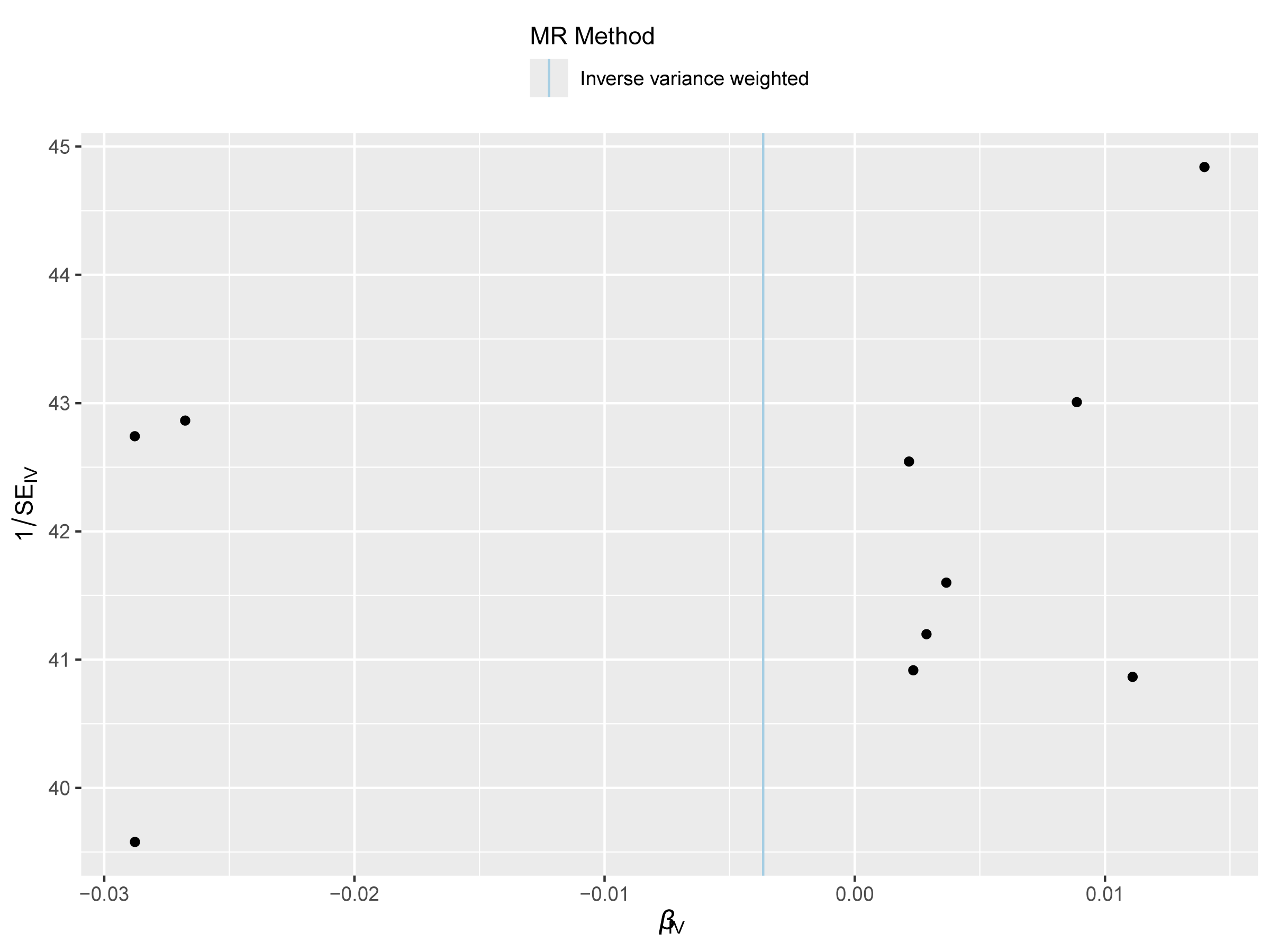

Supplement: Supplementary file 2 [file Data_Sheet_1.zip › supplementary figures/Figure S6 A.tif]

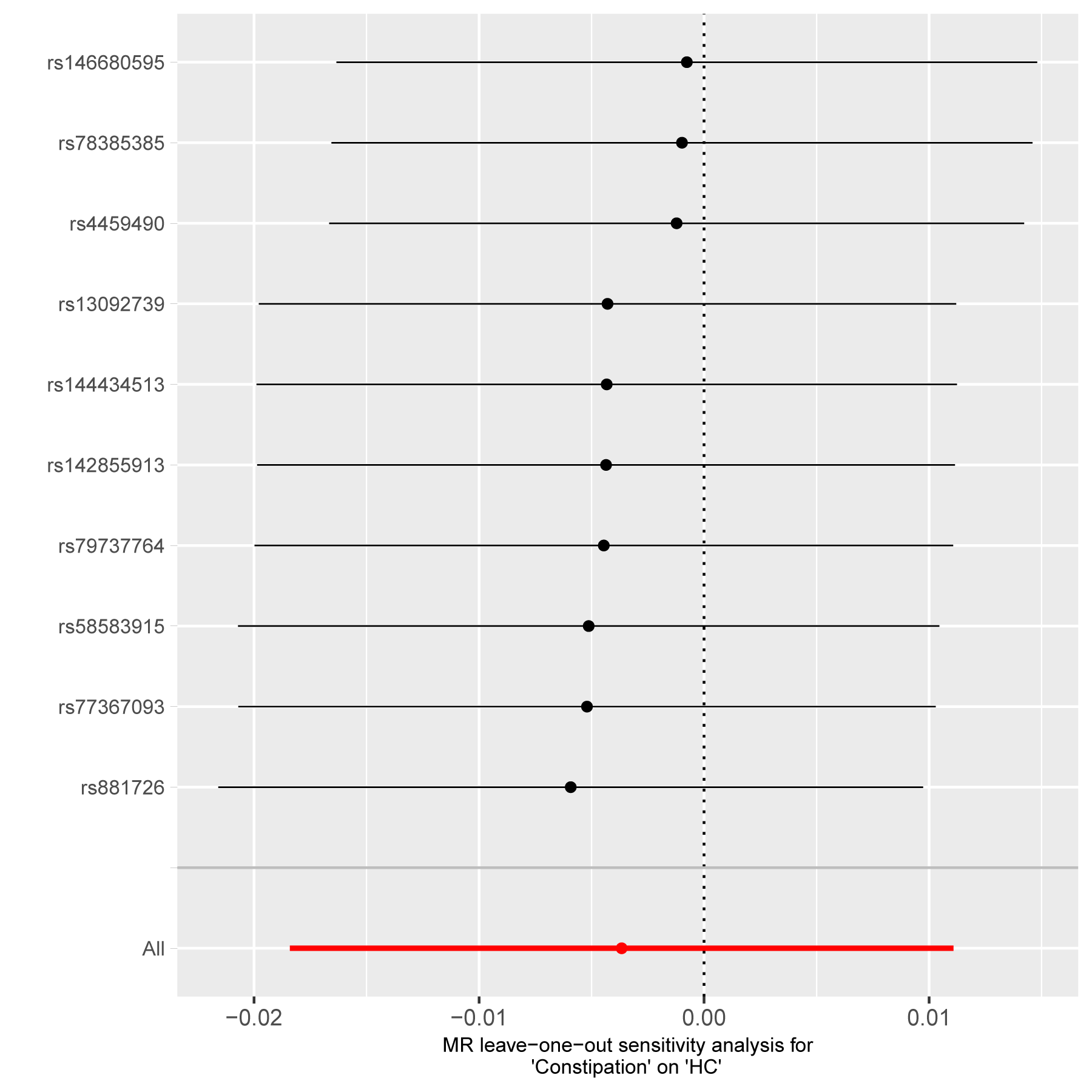

Supplement: Supplementary file 2 [file Data_Sheet_1.zip › supplementary figures/Figure S6 B.tif]

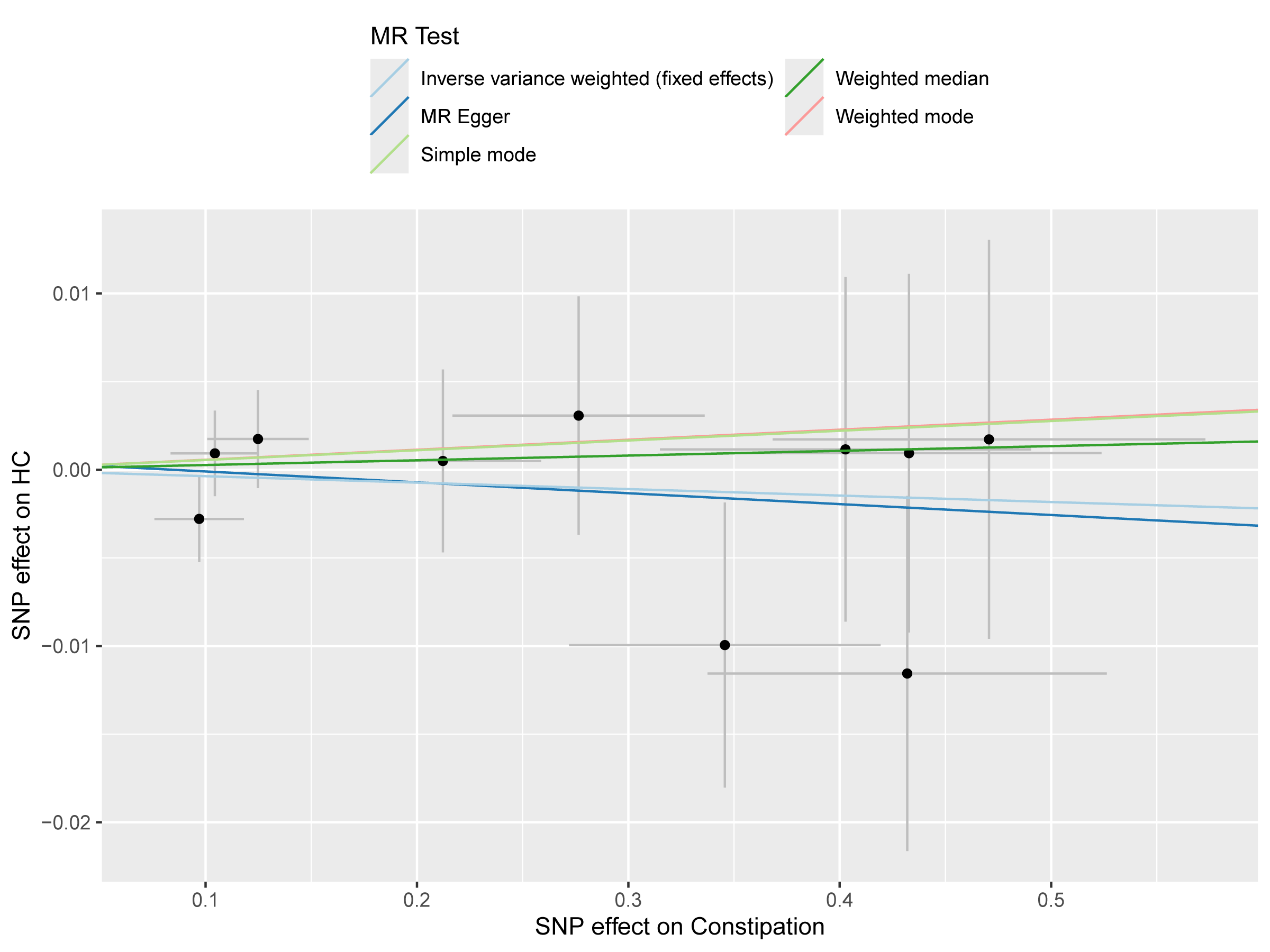

Supplement: Supplementary file 2 [file Data_Sheet_1.zip › supplementary figures/Figure S6 C.tif]

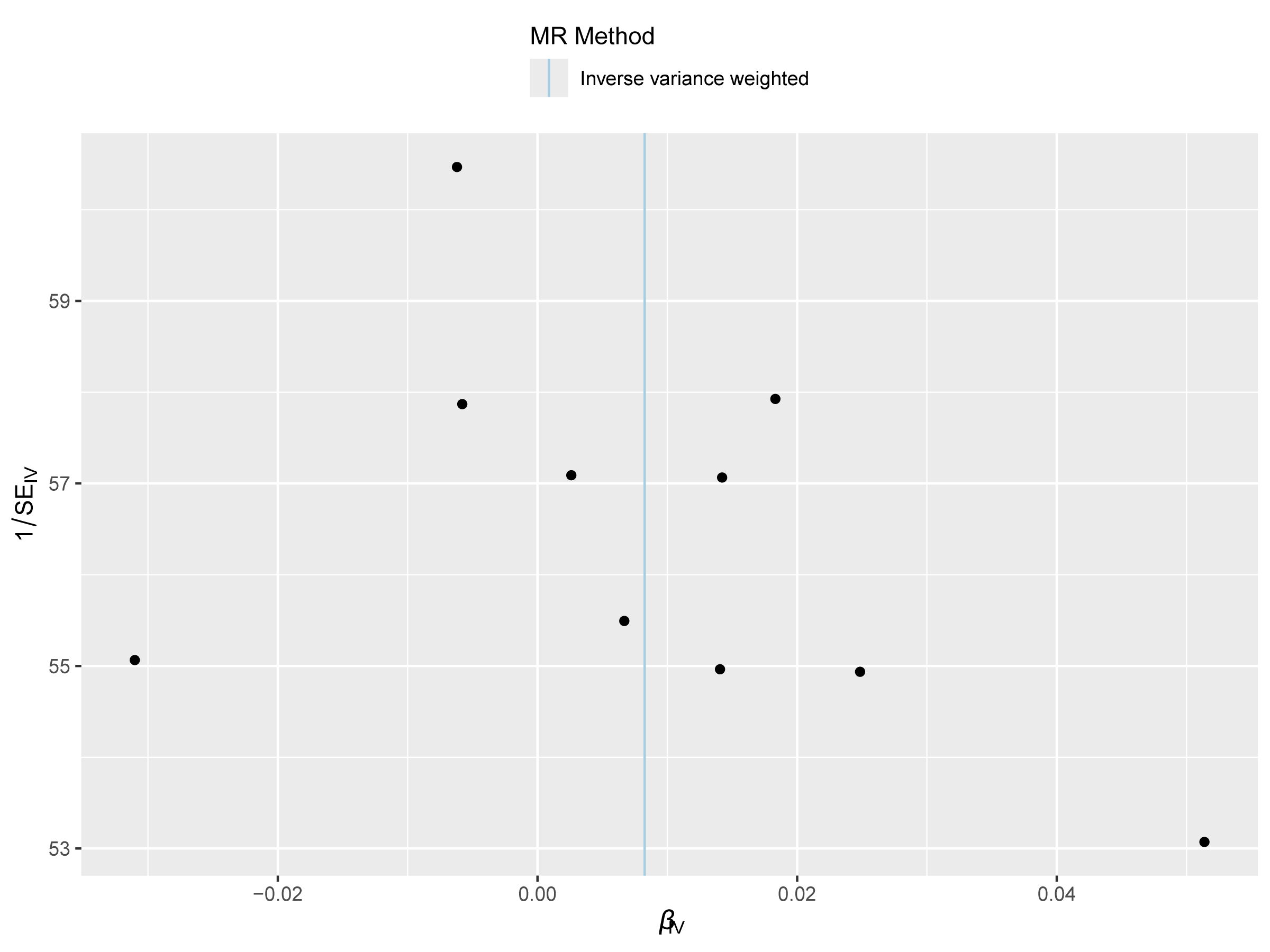

Supplement: Supplementary file 2 [file Data_Sheet_1.zip › supplementary figures/Figure S7 A.tif]

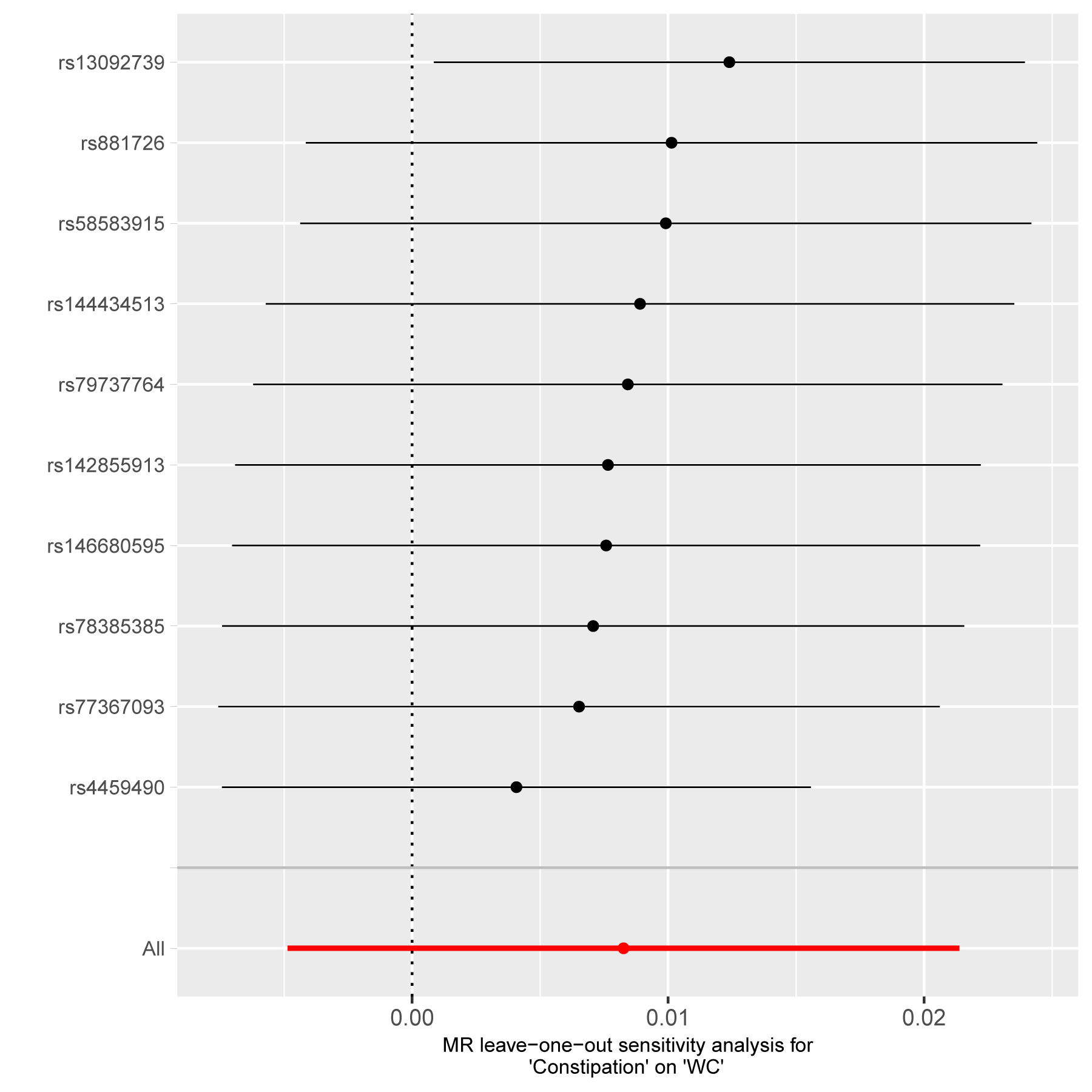

Supplement: Supplementary file 2 [file Data_Sheet_1.zip › supplementary figures/Figure S7 B.tif]

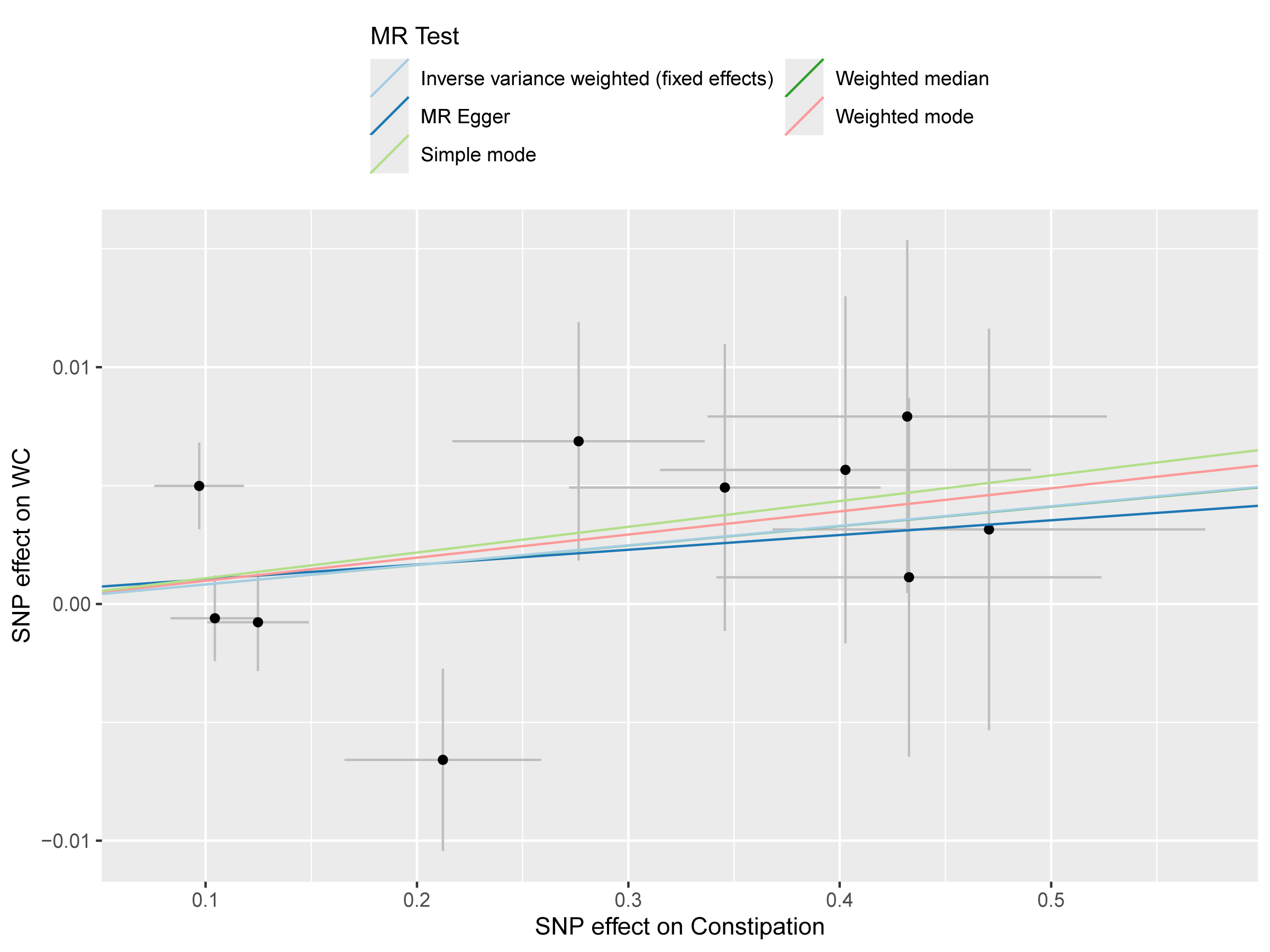

Supplement: Supplementary file 2 [file Data_Sheet_1.zip › supplementary figures/Figure S7 C.tif]

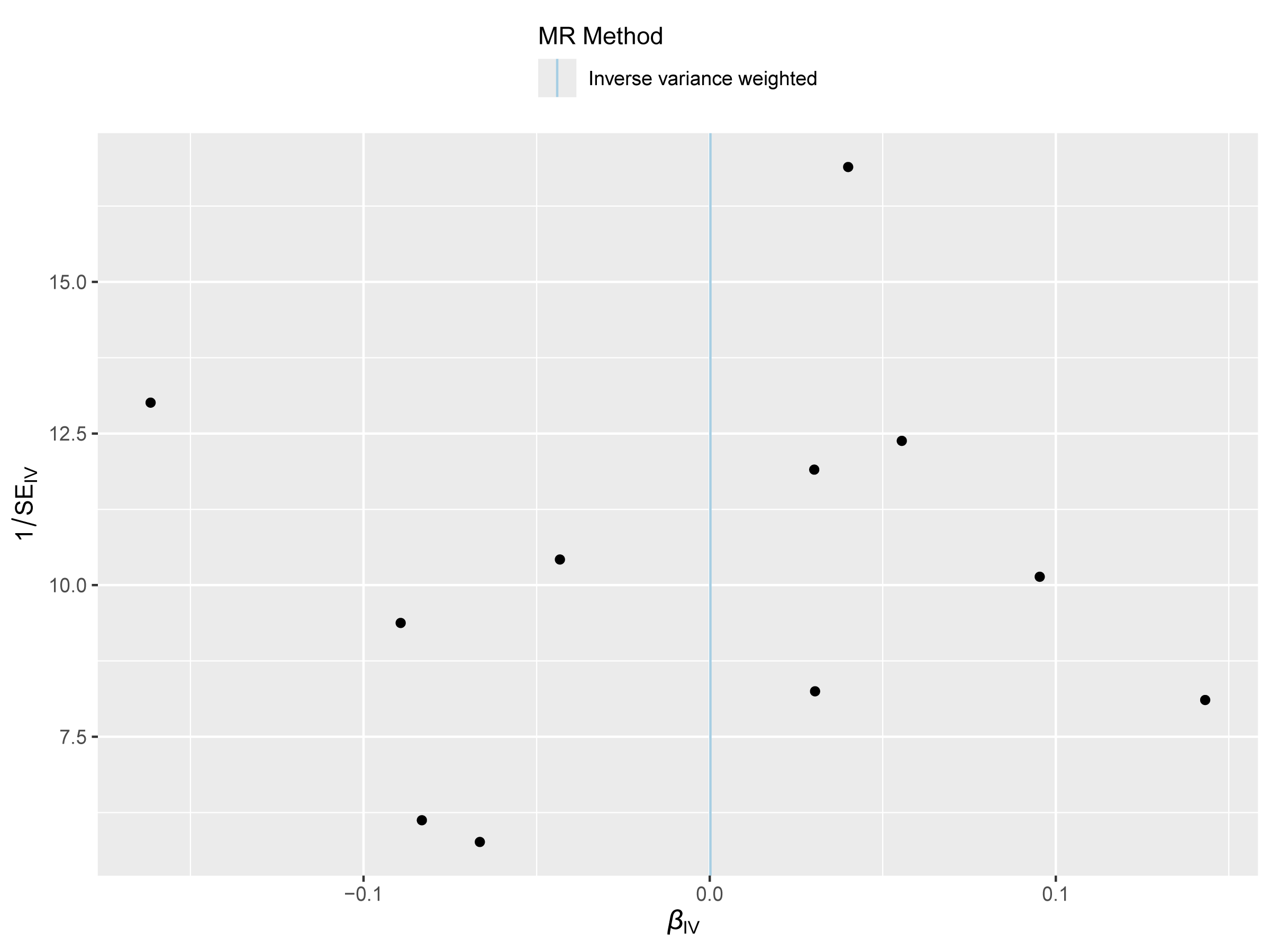

Supplement: Supplementary file 2 [file Data_Sheet_1.zip › supplementary figures/Figure S8 A.tif]

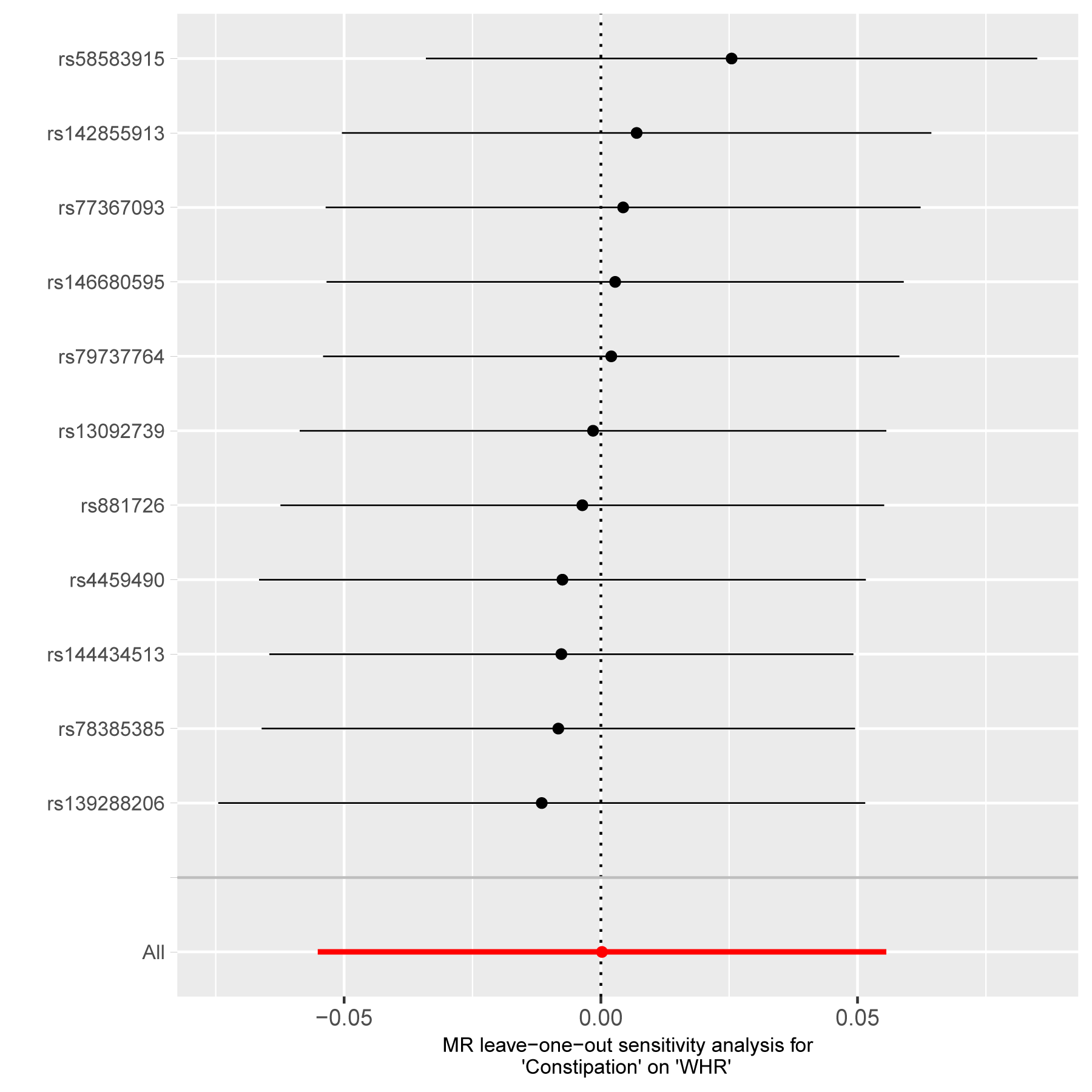

Supplement: Supplementary file 2 [file Data_Sheet_1.zip › supplementary figures/Figure S8 B.tif]

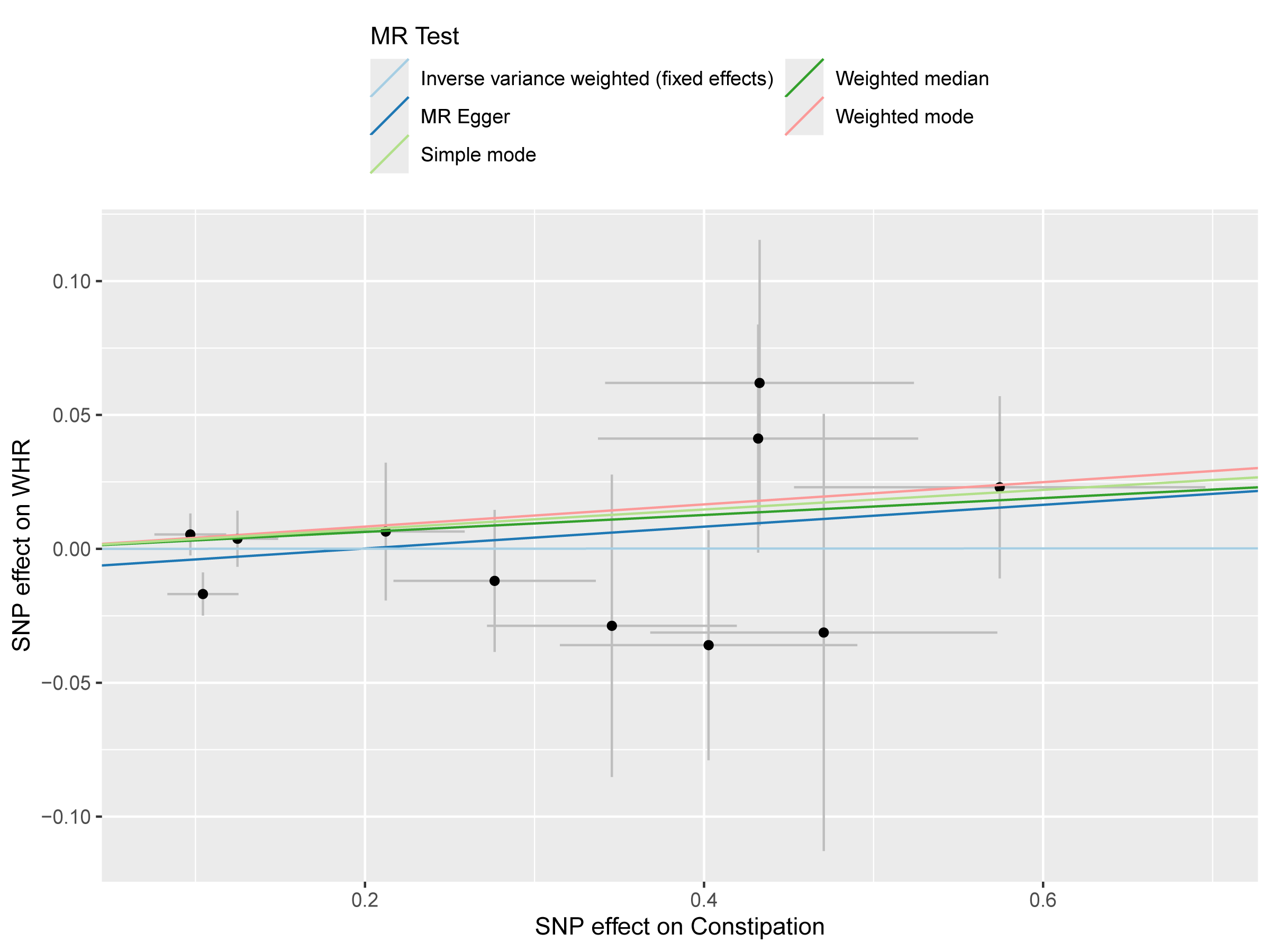

Supplement: Supplementary file 2 [file Data_Sheet_1.zip › supplementary figures/Figure S8 C.tif]

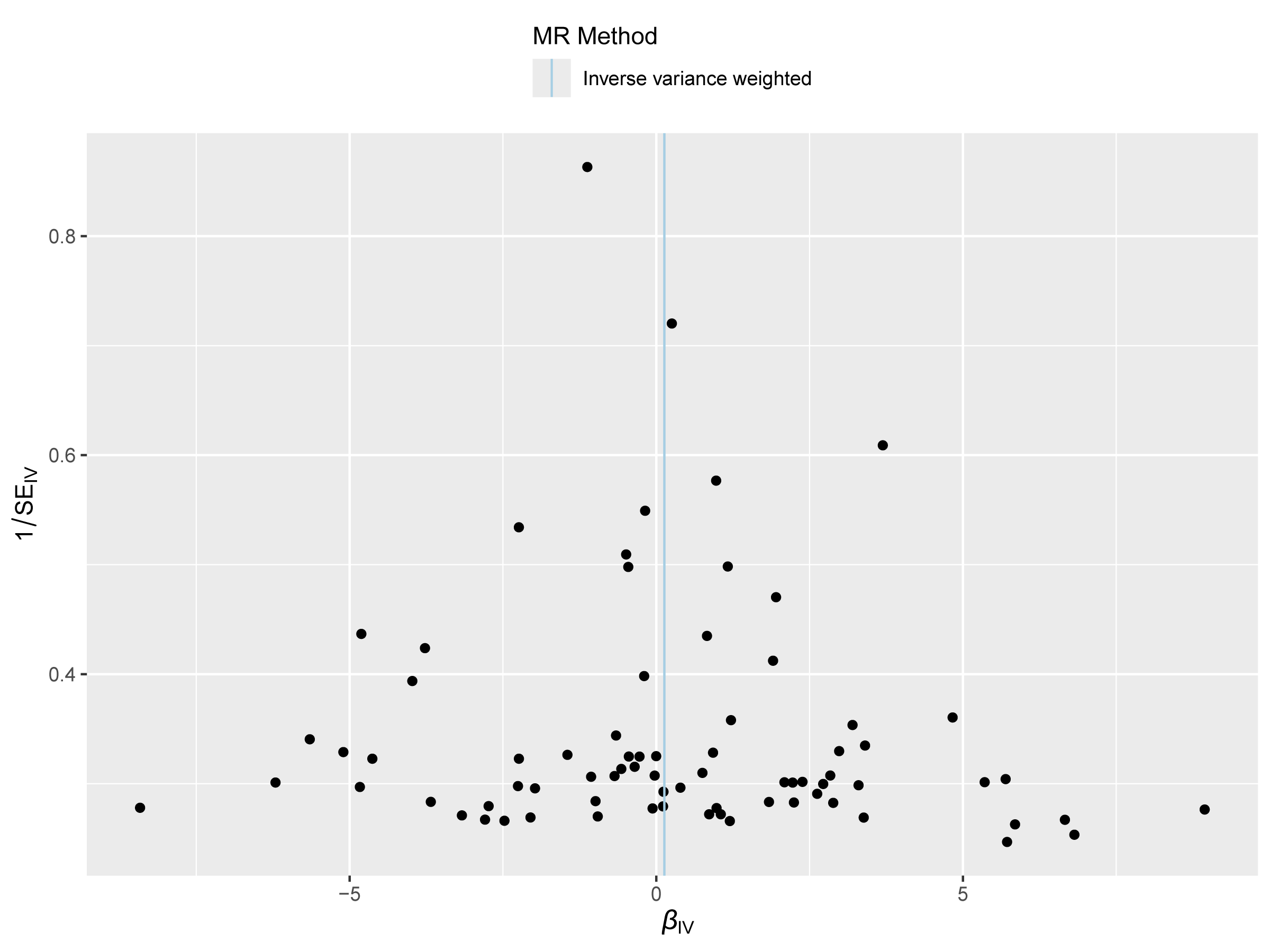

Supplement: Supplementary file 2 [file Data_Sheet_1.zip › supplementary figures/Figure S9 A.tif]

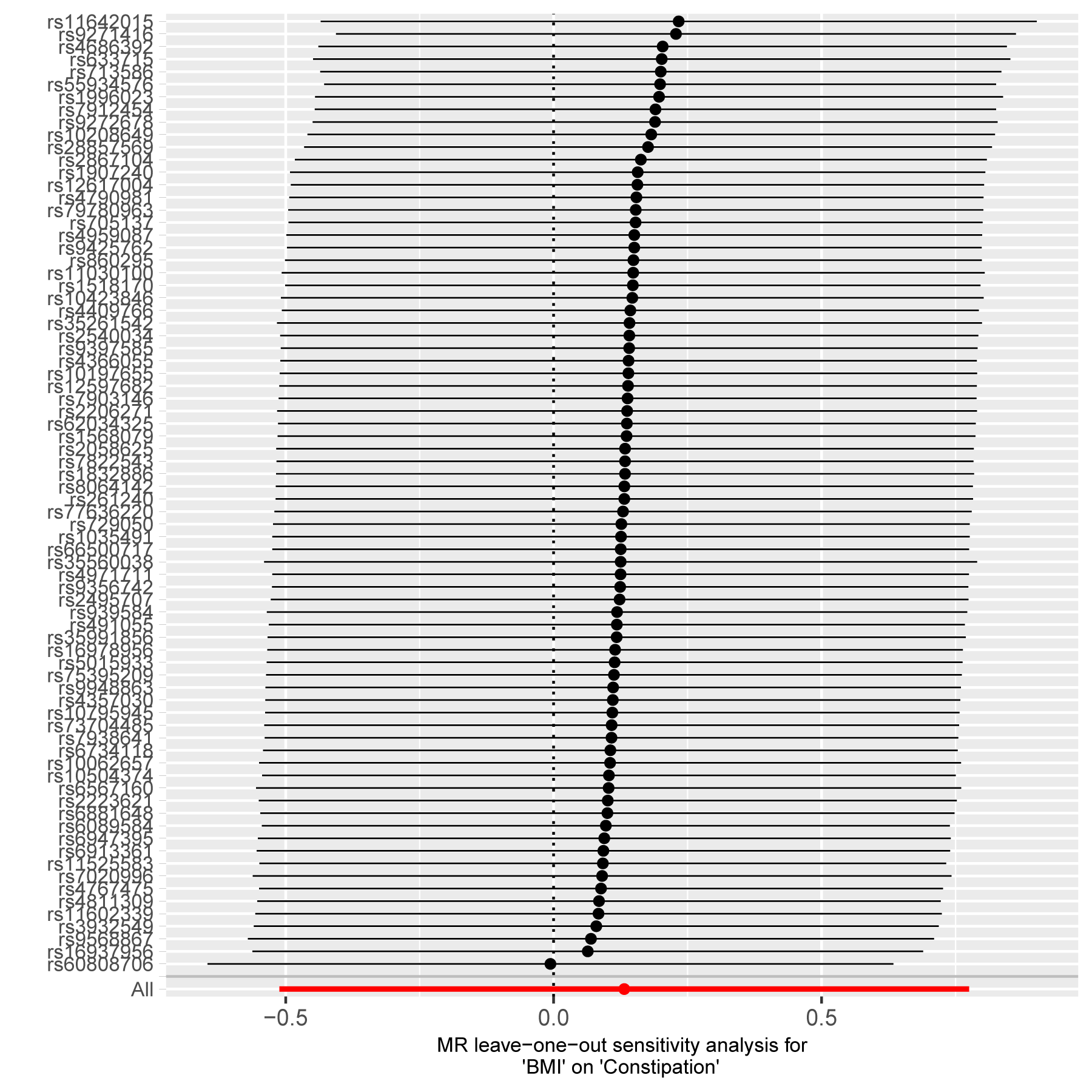

Supplement: Supplementary file 2 [file Data_Sheet_1.zip › supplementary figures/Figure S9 B.tif]

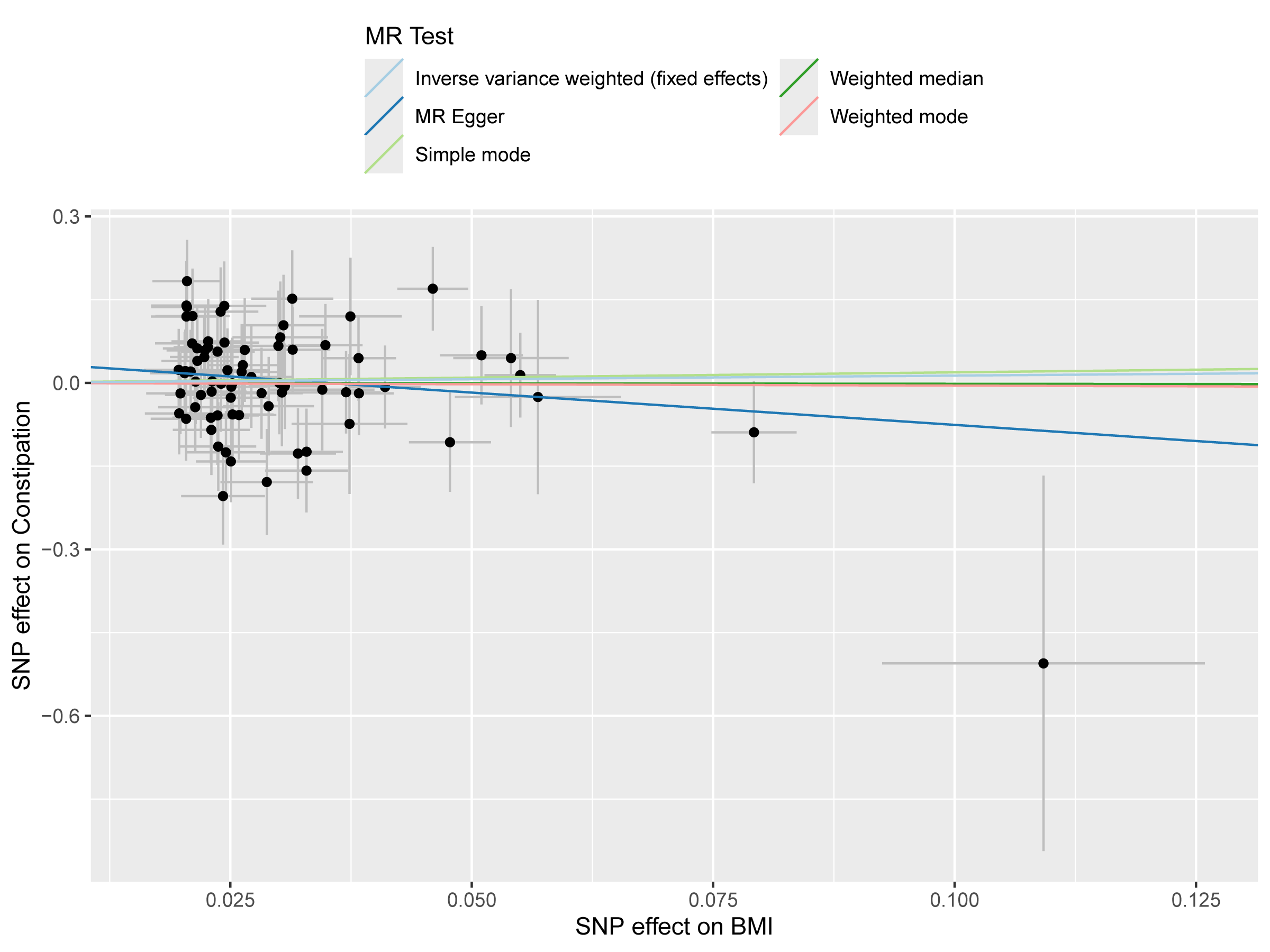

Supplement: Supplementary file 2 [file Data_Sheet_1.zip › supplementary figures/Figure S9 C.tif]
